# Inter- and intratumoral proteomics and glycosaminoglycan characterization of ALK rearranged lung adenocarcinoma tissues: a pilot study

**DOI:** 10.1038/s41598-023-33435-1

**Published:** 2023-04-17

**Authors:** Mirjam Balbisi, Simon Sugár, Gitta Schlosser, Beáta Szeitz, János Fillinger, Judit Moldvay, László Drahos, A. Marcell Szász, Gábor Tóth, Lilla Turiák

**Affiliations:** 1 MS Proteomics Research Group, Research Centre for Natural Sciences, Magyar Tudósok körútja 2., Budapest, 1117 Hungary; 2 Doctoral School of Pharmaceutical Sciences, Semmelweis University, Üllői út 26., Budapest, 1085 Hungary; 3 MTA-ELTE Lendület Ion Mobility Mass Spectrometry Research Group, Eötvös Loránd University, Pázmány Péter sétány 1, Budapest, 1117 Hungary; 4 Division of Oncology, Department of Internal Medicine and Oncology, Semmelweis University, Üllői út 26., Budapest, 1085 Hungary; 5 Department of Pathology, National Korányi Institute of Pulmonology, Korányi Frigyes út 1., Budapest, 1121 Hungary; 6 1st Department of Pulmonology, National Korányi Institute of Pulmonology, Korányi Frigyes út 1., Budapest, 1121 Hungary

**Keywords:** Lung cancer, Glycomics, Proteomics

## Abstract

Lung cancer is one of the most common types of cancer with limited therapeutic options, therefore a detailed understanding of the underlying molecular changes is of utmost importance. In this pilot study, we investigated the proteomic and glycosaminoglycan (GAG) profile of ALK rearranged lung tumor tissue regions based on the morphological classification, mucin and stromal content. Principal component analysis and hierarchical clustering revealed that both the proteomic and GAG-omic profiles are highly dependent on mucin content and to a lesser extent on morphology. We found that differentially expressed proteins between morphologically different tumor types are primarily involved in the regulation of protein synthesis, whereas those between adjacent normal and different tumor regions take part in several other biological processes (e.g. extracellular matrix organization, oxidation–reduction processes, protein folding) as well. The total amount and the sulfation profile of heparan sulfate and chondroitin sulfate showed small differences based on morphology and larger differences based on mucin content of the tumor, while an increase was observed in both the total amount and the average rate of sulfation in tumors compared to adjacent normal regions.

## Introduction

Lung cancer is one of the most prevalent cancer types, and is responsible for the most cancer-related death events with an estimated 1.8 million events worldwide in 2021^1^. The most common form is non-small cell lung cancer (NSCLC), which is accountable for approximately 80–85% of all lung cancer cases^1^. Around 60% of NSCLCs are adenocarcinoma, 30–35% are squamous cell carcinoma and there are less frequent subtypes as well, such as large cell mixed or large cell neuroendocrine types^2^. The most common genetic abnormalities responsible for the development of adenocarcinoma affect the *KRAS*, *EGFR*, *ALK*, *RET*, *ROS1*, *BRAF*, *HER2*, and *MET* genes^1^. Anaplastic lymphoma kinase (ALK) protein belongs to the tyrosine kinase protein family and—just like the other members of the family—is responsible for cell growth. About 4% of people with NSCLC have a rearrangement in the *ALK* gene on chromosome 2, leading to a fusion of *ALK* with another gene^3^. The most common *ALK* fusion partner is *EML4*, which results in the production of the EML4-ALK fusion protein and consequently, leads to the activation of ALK and its downstream signaling pathways, such as RAS and ERK^3,4^. Several ALK inhibitor drugs are available for the treatment of this cancer type, but the majority of patients develop resistance to these drugs over time^5^.

The histological structure of tumors is very diverse. Papillary tumors have a glove-finger-shaped arrangement^6^, while tubular tumors contain many connective tissue elements and are characterized by a tubular shape^7^. However, the most common type is solid tumor, which is a non-cystic mass of tissue, such as carcinoma, lymphoma and sarcoma^8^. In general, lung cancer patients with papillary or solid tumors have a worse prognosis than those with tubular one^9^. In most cases, however, the lung tumor is not uniform but contains regions belonging to several different morphological classes^10^.

Mucins are high molecular weight, highly *O*-glycosylated proteins known for their gel-forming ability, which play an important role in the signal transduction process. Overexpression of these proteins, in particular the MUC1 protein, has been observed in several cancer types^11^. Mucin-type *O*-glycans are glycan chains, whose first building unit is an *N*-acetylgalactosamine. The synthesis of mucin-type *O*-glycans is catalyzed by GalNAc transferase (GALNT) enzymes. On the other hand, stroma is the local environment of the cells, the part of the tissue or organ that does not have the specific functions of the organ. The largest part of this is the extracellular matrix (ECM), which contains a large amount of proteoglycans (PGs). These PGs provide viscoelastic properties and help the proper collagen organization in the ECM^12^.

One of the most common post-translational modifications (PTMs) considerably affecting the structure and function of proteins is glycosylation^13^. Proteoglycans are formed by the attachment of sulfated linear polysaccharides (glycosaminoglycans, GAGs) to the core protein^14^. PGs are mostly found on the cell surface and in the ECM. These molecules can interact with cells and many ECM molecules, thus playing an important role in signal transduction, cell growth, cell division, and many more pathways involved in cancer progression^15^. Chondroitin sulfate (CS) GAGs are built up of alternating glucuronic acid and *N*-acetylgalactosamine units, while heparan sulfate (HS) GAGs are built up of glucuronic acid/iduronic acid and *N*-acetylglucosamine monosaccharides. Both proteins and GAG chains are usually analyzed using a bottom-up approach, i.e. proteins are digested into peptides and GAG chains into disaccharide building blocks, and the resulting components are analyzed by ultra-high performance liquid chromatography coupled to tandem mass spectrometry (UHPLC-MS/MS)^16,17^. Proteins are subjected to differential expression and pathway analyses, and the GAG chains are characterized by their abundance and sulfation characteristics among the studied samples.

Nowadays, most of the proteomics-based studies aim to identify potential diagnostic and prognostic biomarkers in tissues and in liquid biopsies for various diseases while proteomics is also a state-of-the-art methodology for revealing dysregulated biological pathways in several diseases^18,19^. Thus, many studies are available on potential biomarkers in lung cancer and its subtypes as well as on the proteomic characterization of lung cancer, e.g. comparing NSCLC or adenocarcinoma tissues and adjacent normal regions, or small cell lung cancer and the main subtypes of NSCLC^20–23^. To date, only proteogenomic analysis of a few ALK gene rearrangement cases has been reported in the literature^24^, and detailed proteomic characterization of tumors carrying this gene alteration has not been performed. Despite the fact that GAG-omic analysis of tissues is still an emerging field, the role of PGs and GAGs, and the alteration of sulfation pattern have been described in many cancer types, including lung cancer^25–29^. As the composition of the proteome and glycome are strongly interrelated with each other, performing integrated proteomics and GAG-omics may help to map the ECM- and signaling-related changes in detail.

The morphological structure of the tumors along with mucin and stromal content inherently have a major impact on the proteoand GAG-omic profile of the tumor and adjacent regions. In the present study, we aimed to characterize the protein and GAG (CS and HS) profile of ALK rearranged lung tumor regions. We compared distinct tumor and adjacent normal regions based on the morphological classification (adjacent normal; papillary, tubular and solid tumor), mucin and stromal content. Mapping the intratumoral differences between the given tissue regions with distinct characteristics may give a better insight into the biological processes underlying tumor formation and progression.

## Results

**Figure 1:**
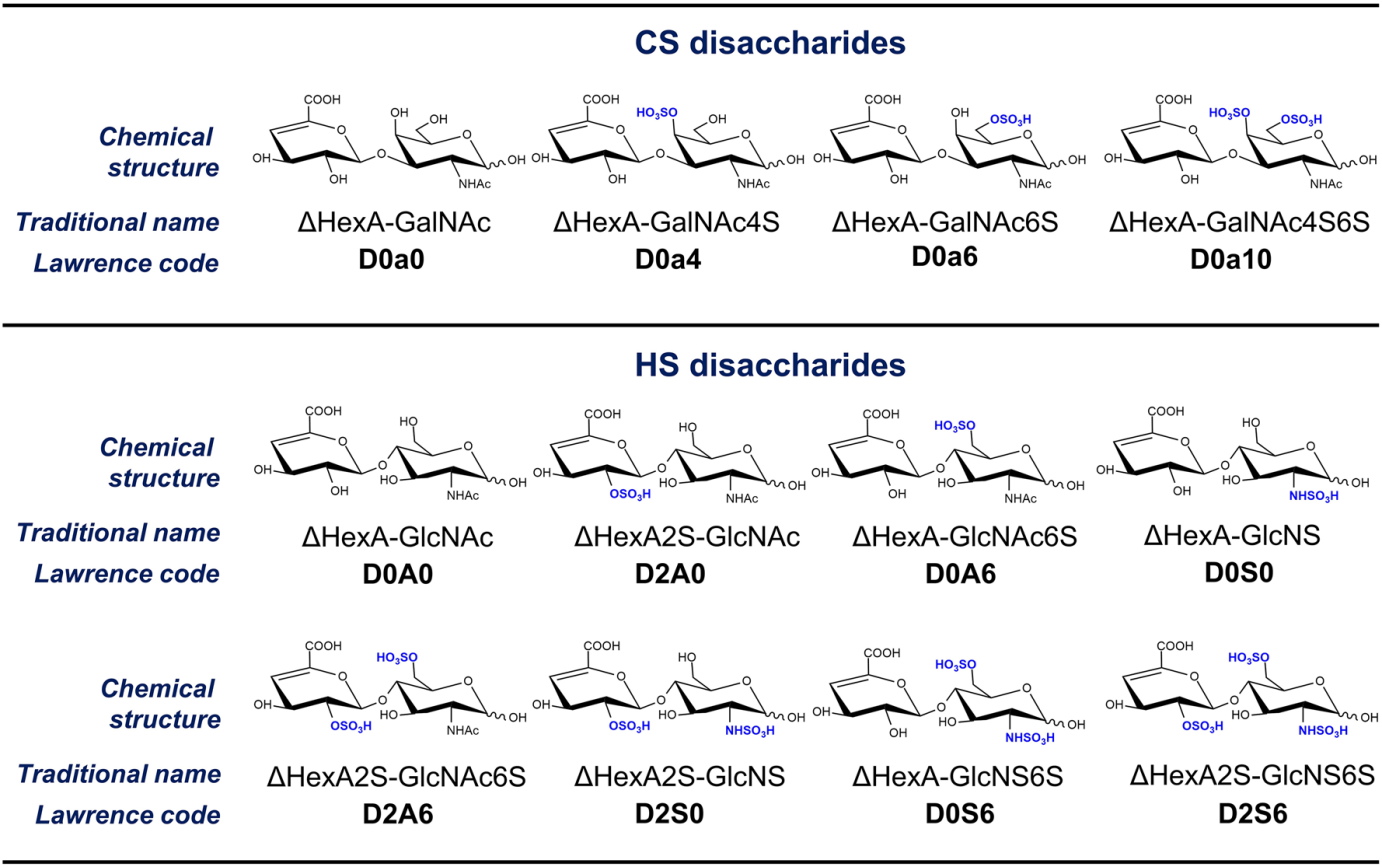
Lawrence code designation and structural formula of disaccharides produced during enzymatic digestion of GAG chains.

A total of 22 tissue regions from 7 patients have been investigated. The tumor regions were classified based on morphological classification, mucin content and stromal content. Samples from the adjacent normal regions were also analyzed. Thus, the morphological classification’s groups were solid tumor (*n* = 10), tubular tumor (*n* = 3), papillary tumor (*n* = 5) and adjacent normal lung (*n* = 4). Regions with medium (M2, *n* = 4) and high amount (M3, *n* = 5) of mucin as well as regions with low (S1, *n* = 5), medium (S2, *n* = 5) and high amount (S3, *n* = 7) of stroma were compared. The properties of the individual regions are summarized in Table 1. First, label-free quantitative proteomics experiments were performed; proteins were tested for expression differences across the sample groups and the biological function of differentially expressed proteins was visualized via protein interaction networks. The levels of individual proteoglycan core proteins were also compared. Next, chondroitin sulfate (CS) and heparan sulfate (HS) glycosaminoglycan chains were investigated by disaccharide analysis after bacterial lyase digestion. Herein, the relative abundance and total amount of the disaccharide building blocks, the average rate of sulfation, and derived sulfation characteristics (the ratio of D0a6/D0a4 monosulfated disaccharides, 6S/4S ratio hereinafter for CS; and the ratio of *N*- and *O*-sulfation for HS) are discussed, while statistical results observed for GAG disaccharides are summarized in Supplementary Table S1. The GAG disaccharides analyzed in the study are represented in Fig. 1. The data collection and analyses are described in detail in “Methods” chapter.

**Table 1:** Characterization of the investigated regions by morphology, mucin content and stromal content. Scores mean the following: 1—low, 2—medium, 3—high amount.

Region number	Patient number	Morphological classification	Mucin (1–3)	Stroma (1–3)
1	1/A	Tubular	3	3
2	1/B	Tubular	3	3
3	2/A	Solid	2	2
4	2/B	Normal	–	–
5	3/A	Normal	–	–
6	3/B	Normal	–	–
7	3/C	Tubular	2	1
8	3/D	Papillary	2	1
9	3/E	Papillary	2	3
10	4/A	Papillary	3	2
11	4/B	Papillary	3	3
12	4/C	Papillary	3	2
13	5/A	Solid	–	2
14	5/B	Solid	–	2
15	5/C	Solid	–	3
16	5/D	Solid	–	–
17	6/A	Solid	–	1
18	6/B	Solid	–	1
19	6/C	Solid	–	1
20	7/A	Solid	1	3
21	7/B	Solid	–	3
22	7/C	Normal	–	–

### Comparison of regions with different morphological classifications

In the shotgun proteomics experiments, an average of 651 proteins per region and a total of 3411 proteins were identified, out of which a total of 2092 proteins were quantified.

In the quantitative proteomic analysis, 250, 380, and 541 differentially expressed proteins were observed in the pairwise comparisons of adjacent normal regions with tubular, papillary, and solid tumor morphological regions, respectively. Focusing on tumor regions, 40 proteins were differentially expressed between papillary and tubular, 83 between solid and tubular, and 199 between papillary and solid tumor morphological regions.

**Figure 2:**
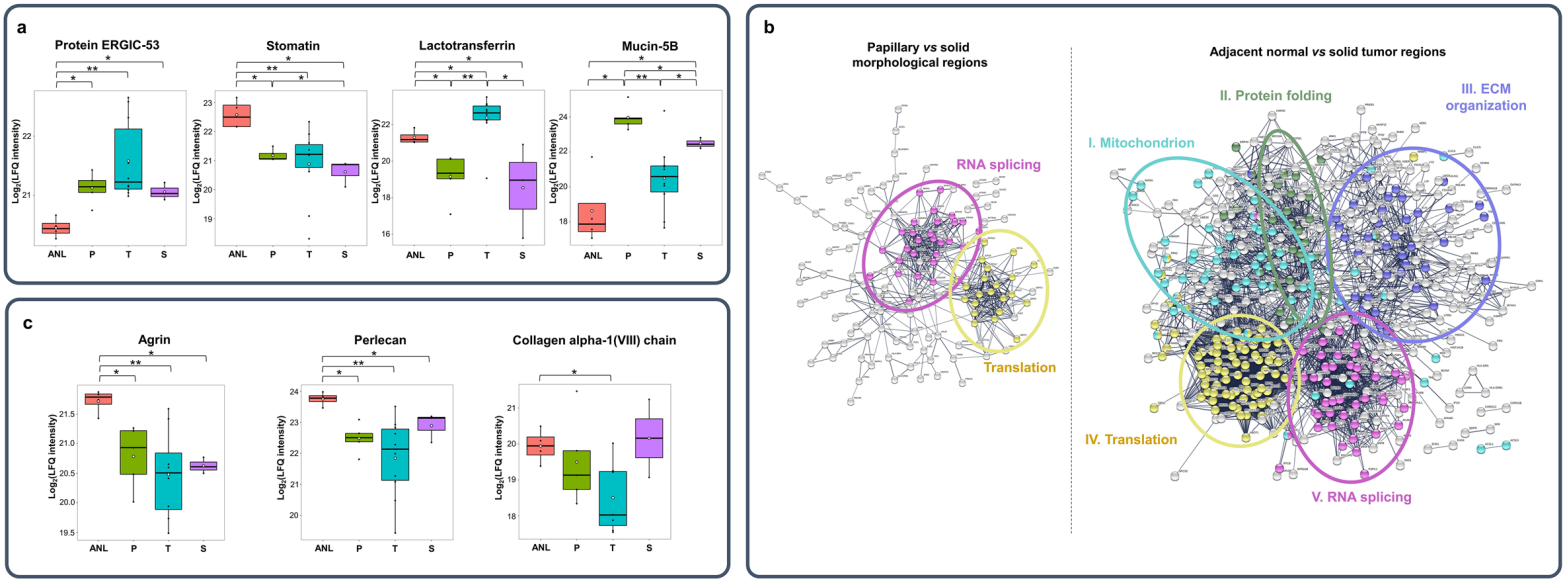
Proteomics differences of lung tissue regions with different morphological classifications (*ANL* adjacent normal lung, *P* papillary tumor, *T* tubular tumor, *S* solid tumor). (**a**) Examples for differentially expressed proteins, (**b**) protein interaction networks comparing papillary tumors with solid tumors and ANL regions with solid tumor ones, (**c**) differentially expressed proteoglycan core proteins (*p < 0.05 (or 0.057 for ANL vs T); **p < 0.01).

The lists of differentially expressed proteins in each comparison are listed in Supplementary Table S2. For example, protein ERGIC-53 was overexpressed, while stomatin was underexpressed in all tumor regions compared to adjacent normal ones with fold-changes (FCs) of 1.5–2.2 and 0.26–0.38, respectively (Fig. 2a). Lactotransferrin was differentially expressed between solid and other tumor types, while mucin-5B showed significant difference between all the three tumor groups.

To reveal the roles of the differentially expressed proteins, protein interaction networks were constructed with STRING GO webserver. Counts in network, strengths, and false discovery rates for the examined biological processes and localizations are shown in Supplementary Table S3 for all the comparisons performed.

Proteins differentially expressed between papillary and solid regions were mostly involved in protein synthesis and could be divided into two clusters taking part in translation (24 of 336) and RNA splicing (29 of 396) (Fig. 2b). These proteins were mostly underexpressed in papillary tumors relative to solid ones.

Between adjacent normal and solid tumor regions, differentially expressed proteins could be divided into 5 clusters. Cluster I represents proteins occurring in mitochondria (69 of 1611), cluster II indicates proteins involved in protein folding (33 of 213), while those in cluster III take part in ECM organization (37 of 338). Members of cluster IV and V are involved in translation (81 of 366) and RNA splicing (48 of 396), thus they both play a role in protein synthesis. Proteins in cluster III were mostly underexpressed, while those in clusters I, II, IV and V were overexpressed in all tumor regions compared to the adjacent normal ones. Protein networks built on differentially expressed proteins between adjacent normal *vs* tubular and papillary tumor groups are shown in Supplementary Fig. S1, highlighting that these proteins are mostly RNA binding proteins or participate in ECM organization or oxidation–reduction processes.

Three heparan sulfate proteoglycan (HSPG) core proteins were identified to be differentially expressed between adjacent normal and tumor tissue regions: agrin, perlecan, and collagen alpha-1 (VIII) chain (Fig. 2c). The abundances of agrin and perlecan were lower in any tumor regions compared to adjacent normal ones, while collagen alpha-1(VIII) chain was underexpressed in solid tumor, with FCs of 0.27–0.55 for all the aforementioned comparisons. Among the various tumor types, no PG core proteins were differentially expressed.

**Figure 3:**
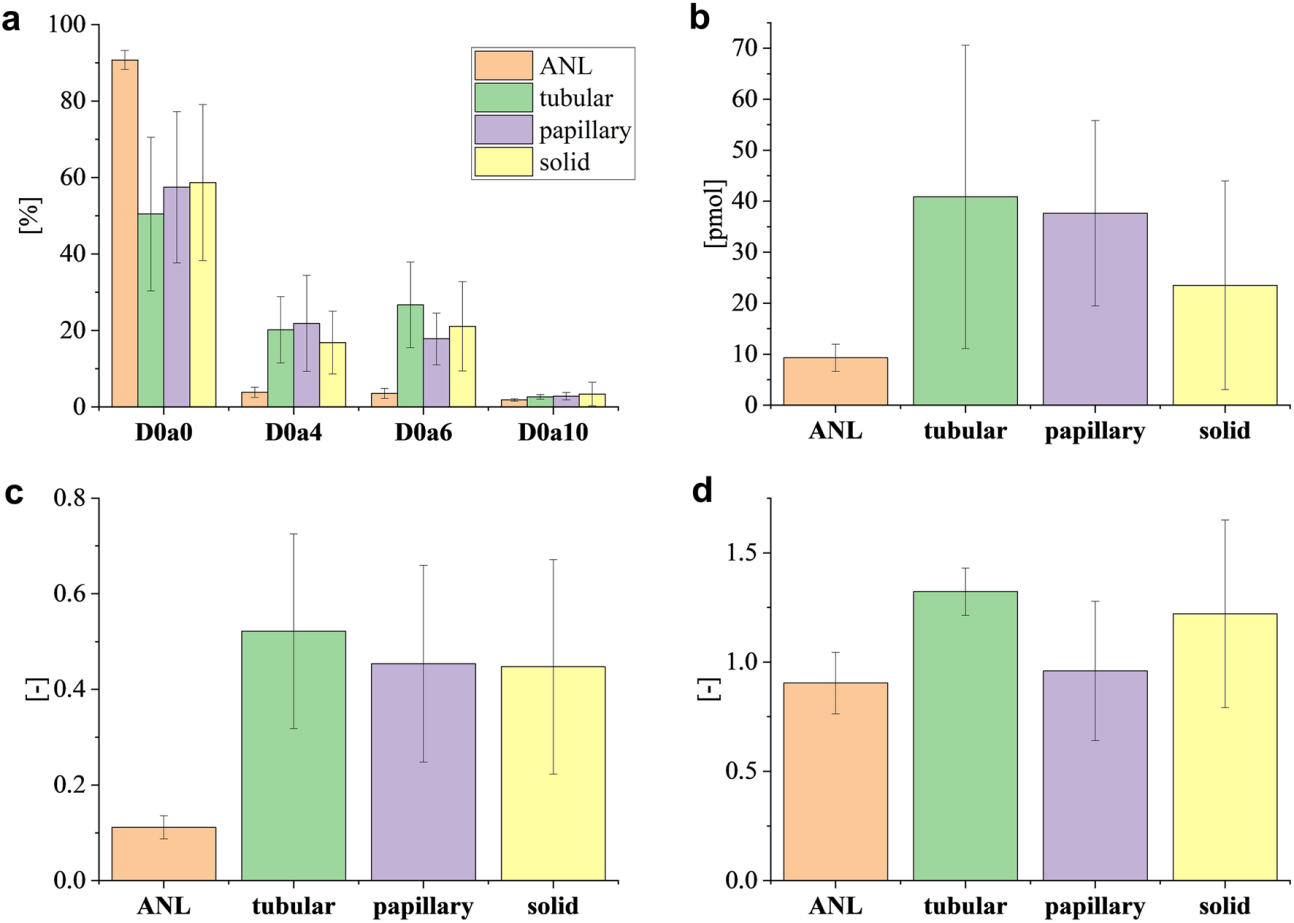
Chondroitin sulfate expression characteristics in lung tissue regions with different morphological classifications (*ANL* adjacent normal lung). (**a**) Relative amount of CS disaccharides (%), (**b**) total amount of CS disaccharides (pmol), (**c**) average rate of CS sulfation, (**d**) 6S/4S ratio. Error bars represent standard deviation.

Next, GAG disaccharide analysis was performed. In adjacent normal regions, the ratio of the non-sulfated D0a0 CS component was 1.5–1.8 times higher than in the distinct tumor sample groups, while all the sulfated components occurred in a higher proportion in the tumor groups (Fig. 3a). Compared to adjacent normal regions, the total CS amount increased by an average of 2.5, 4.0 and 4.4 times in solid, papillary and tubular regions, respectively (Fig. 3b).

The average rates of CS sulfation were 4.0–4.7 times higher in the tumor regions of the tissue sections compared to the adjacent normal regions, regardless of the morphological classification (Fig. 3c). In adjacent normal tissue regions, the 6S/4S ratio was 0.90, and a similar ratio was observed in the papillary regions (Fig. 3d). However, a different dominant sulfation position was present in the tubular and solid regions, with 6S/4S ratios of 1.32 and 1.22, respectively.

**Figure 4:**
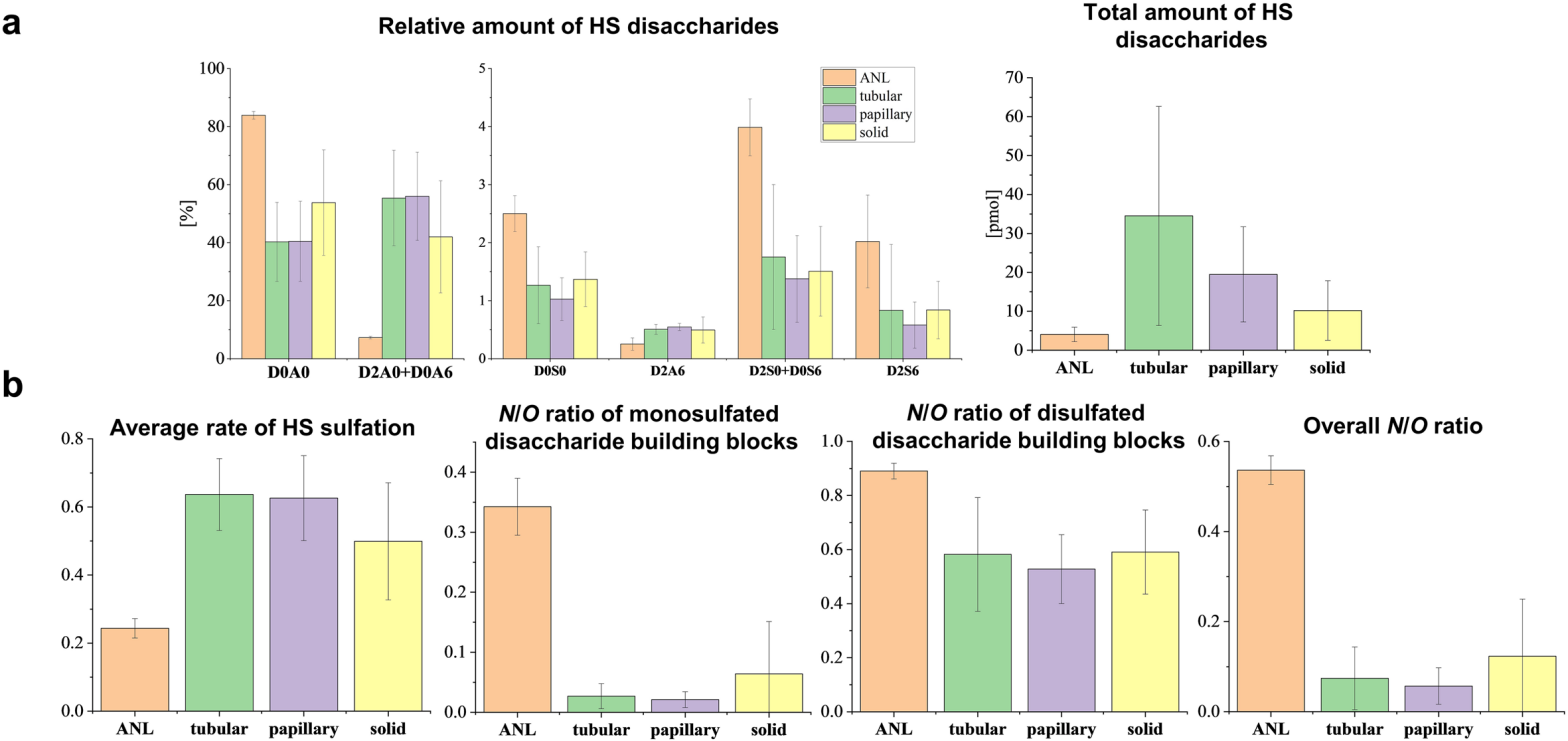
Heparan sulfate expression characteristics in lung tissue regions with different morphological classifications (*ANL* adjacent normal lung). (**a**) Relative amount of HS disaccharide building blocks and total HS amount, (**b**) sulfation characteristics of HS chains. Error bars represent standard deviation.

Next, the expression characteristics of HS chains were investigated. Adjacent normal regions contained a higher portion of D0A0, D0S0, D2S0 + D0S6, and D2S6 HS building blocks, but less *O-*sulfated disaccharides (D2A0 + D0A6 and D2A6) than all the tumor regions (Fig. 4a). We observed remarkable differences in total HS quantities; solid, papillary and tubular morphological regions contained 2.5, 4.8 and 8.5-fold more HS than adjacent normal regions, respectively. The trend in the total amount of HS chains was the same as in the case of CS but larger differences were observed between the tumor sample groups in the case of HS (Fig. 3b vs Fig. 4a).

The average rate of HS sulfation in all tumor regions was 2.0–2.6 times higher than in the adjacent normal ones (Fig. 4b). In contrast, the three tumor groups with different morphological classifications showed only minor differences. The ratio of *N*-sulfation was considerably higher in the adjacent normal tissue than in tumor regions. The monosulfated, disulfated and total *N*/*O* ratios were 0.34, 0.89 and 0.53 in the adjacent normal samples, while those of the distinct tumor types were 1.5–16.2 times lower in all cases.

### Comparison of regions with different mucin content

**Figure 5:**
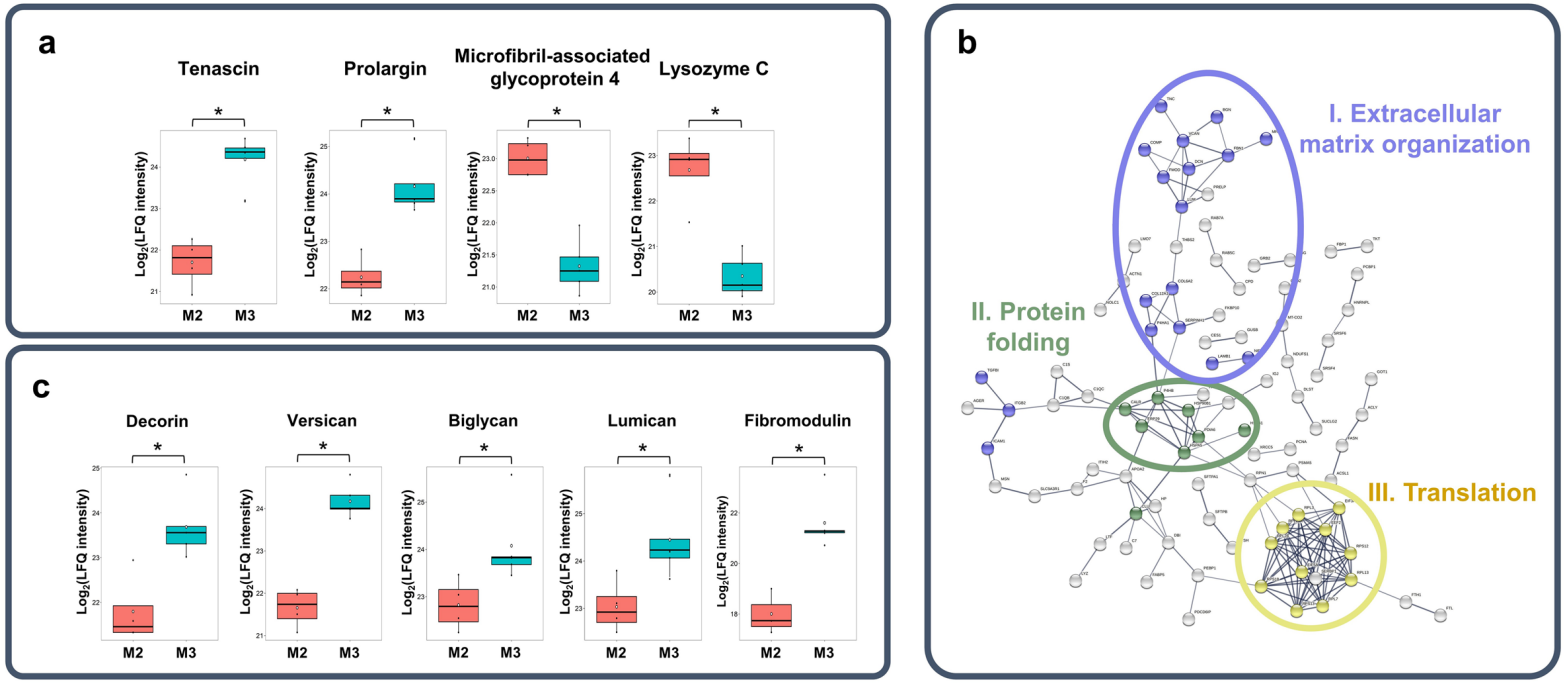
Proteomics differences of lung tissue regions with different mucin content (*M2* medium mucin content, *M3* high mucin content). (**a**) Examples for differentially expressed proteins; (**b**) protein interaction network built on differentially expressed proteins between M2 and M3 tumor regions; (**c**) differentially expressed proteoglycan core proteins (*p < 0.05).

Next, we investigated the molecular differences between regions grouped by their mucin content. In total, 171 proteins were differentially expressed between the regions with medium amount of mucin (M2, *n* = 4) and regions with high amount of mucin (M3, *n* = 5). For example, tenascin and prolargin were overexpressed in M3 regions (with FCs of 5.6 and 3.8, respectively), while the abundance of microfibril-associated glycoprotein 4 and lysozyme C were lower in M3 than in M2 regions (with FCs of 0.31 and 0.20, respectively) (Fig. 5a). Differentially expressed proteins were mainly involved in ECM organization (cluster I, 18 of 338), protein folding (cluster II, 9 of 213) and translation (cluster III, 13 of 366) (Fig. 5b). Proteins of cluster I were typically overexpressed, whereas those of clusters II and III were mostly underexpressed in M3 regions compared to M2 tissues.

A significant overexpression of decorin (CSPG, FC = 3.7), versican (CSPG, FC = 5.7), biglycan (CSPG, FC = 2.4), lumican (KSPG, FC = 2.7) and fibromodulin (KSPG, FC = 12) PG core proteins was observed in M3 regions compared to M2 regions (Fig. 5c).

**Figure 6:**
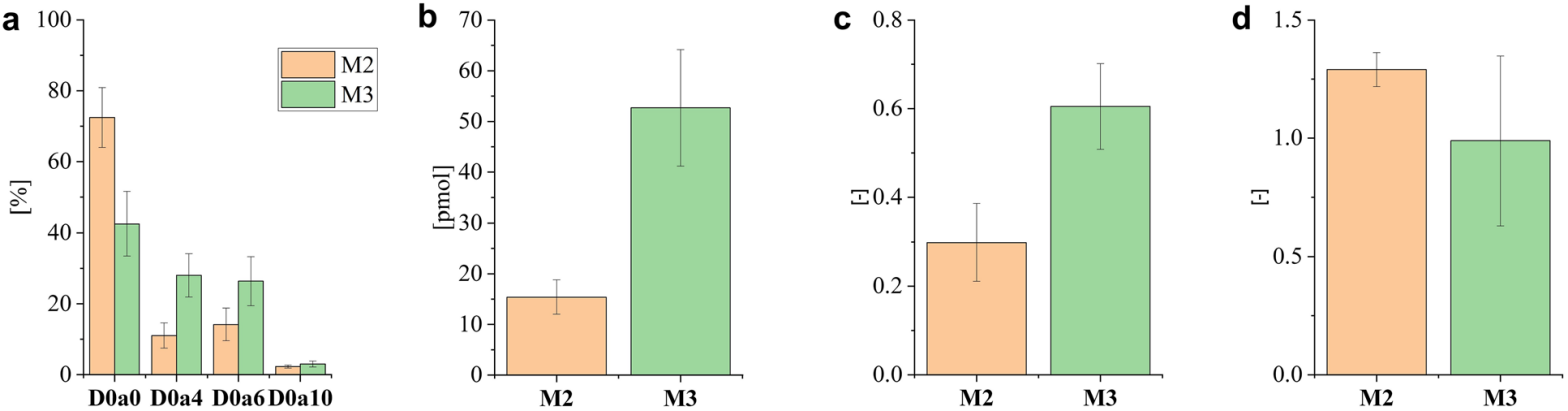
Chondroitin sulfate expression characteristics in lung tissue regions with different mucin content (*M2* medium mucin content, *M3* high mucin content). (**a**) relative amount of CS disaccharides (%), (**b**) total amount of CS disaccharides (pmol), (**c**) average rate of CS sulfation, (**d**) 6S/4S ratio for regions with different mucin content. Error bars represent standard deviation.

Regarding GAG analysis, M2 regions contained 1.7-fold more non-sulfated D0a0 CS disaccharide building blocks than M3 ones, while all sulfated CS components occurred in higher ratio in M3 samples (Fig. 6a). Comparing the total amount of CS, there was a great difference between M2 and M3 regions, as M3 regions contained 3.5-fold more CS (Fig. 6b); this is in accordance with increased expression of CSPG core proteins shown in Fig. 5c. As a result of the change in the sulfation pattern, the average rate of CS sulfation was twice as large in M3 group as in the M2 group (Fig. 6c). The M2 samples contained 1.3-fold more D0a6 than D0a4, while the two monosulfated disaccharides occurred in approximately the same amount in the M3 regions (Fig. 6d).

**Figure 7:**
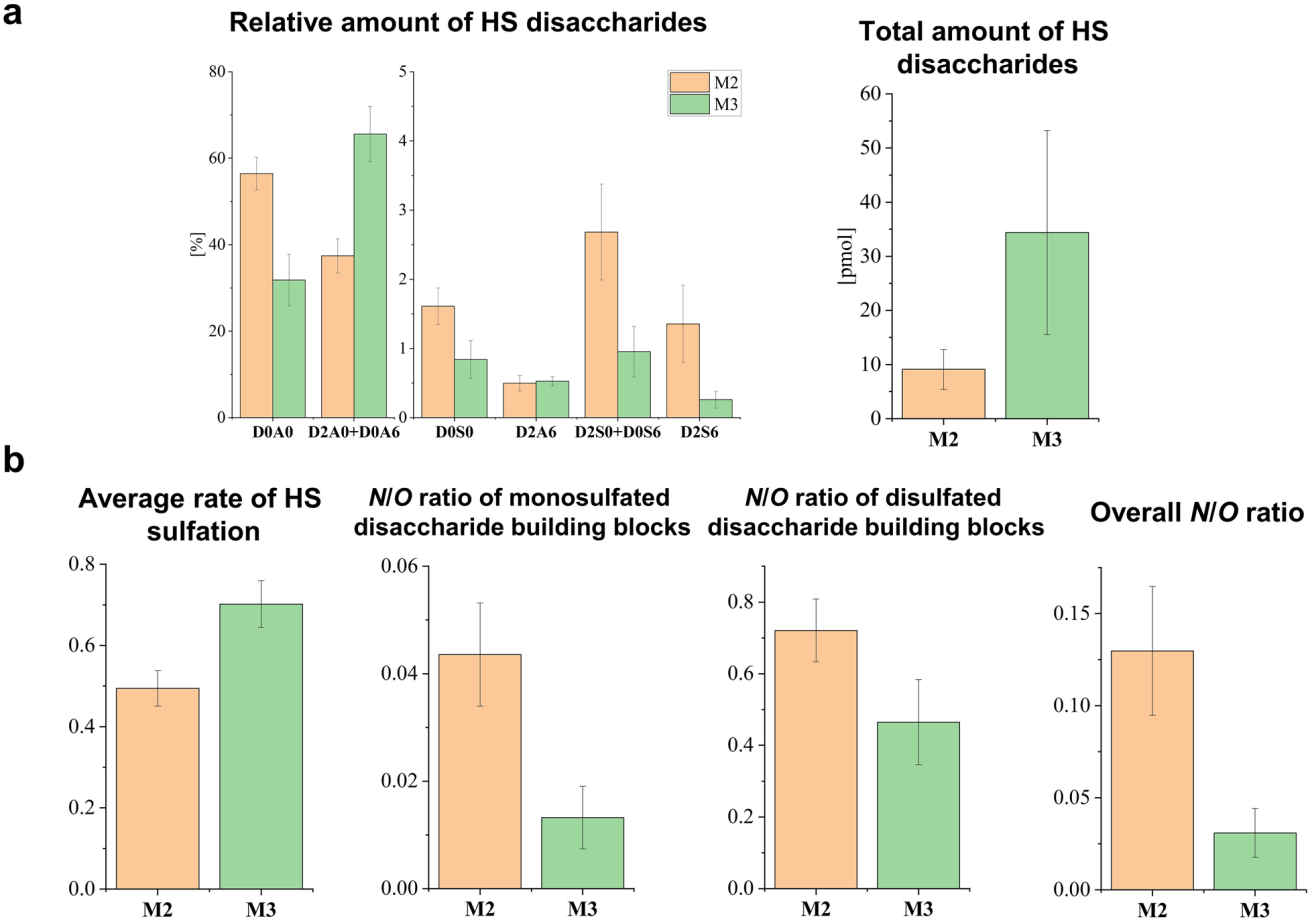
(**a**) Relative and total amount of HS disaccharides, (**b**) sulfation characteristics for regions with different mucin content. Error bars represent standard deviation.

In the case of HS sulfation pattern, M2 samples contained D2A0 + D0A6 component in a lower ratio than M3 samples, while all the other disaccharides were either present in similar amounts in the two sample groups (D2A6), or their proportion was higher in sample group M2 (Fig. 7a). The total HS amount was 3.7-fold higher in the M3 group compared to M2, and the average rate of HS sulfation was 1.4-fold higher in M3 regions (Fig. 7). The *N*/*O* ratios of monosulfated and disulfated components and total *N*/*O* ratio were 1.6–4.2 times less in M3 than in M2 sample group, therefore, an increasing rate of *O-*sulfation was observed with elevated mucin content (Fig. 7b).

### Comparison of regions with different stromal content

Regions containing low (S1, *n* = 5), medium (S2, *n* = 5) and high (S3, *n* = 7) amounts of stroma were compared. 248 proteins were differentially expressed between S1 and S2, 252 between S1 and S3, and 73 between S2 and S3 regions. Most of the differentially expressed proteins were RNA binding proteins or involved in ECM organization and oxidation–reduction processes. Comparing the S1 sample group with S2 and S3 groups, several PG core proteins were differentially expressed (e.g. perlecan, biglycan, decorin, versican, lumican). We observed an elevation in CS and HS total quantities with increasing stromal content, and the overall sulfation of CS chains showed a similar trend as well. Detailed results are shown in the Supplementary material under the section “Comparison of regions with different stromal content” (Figs. S2–S4).

### Hierarchical clustering and principal component analysis

**Figure 8:**
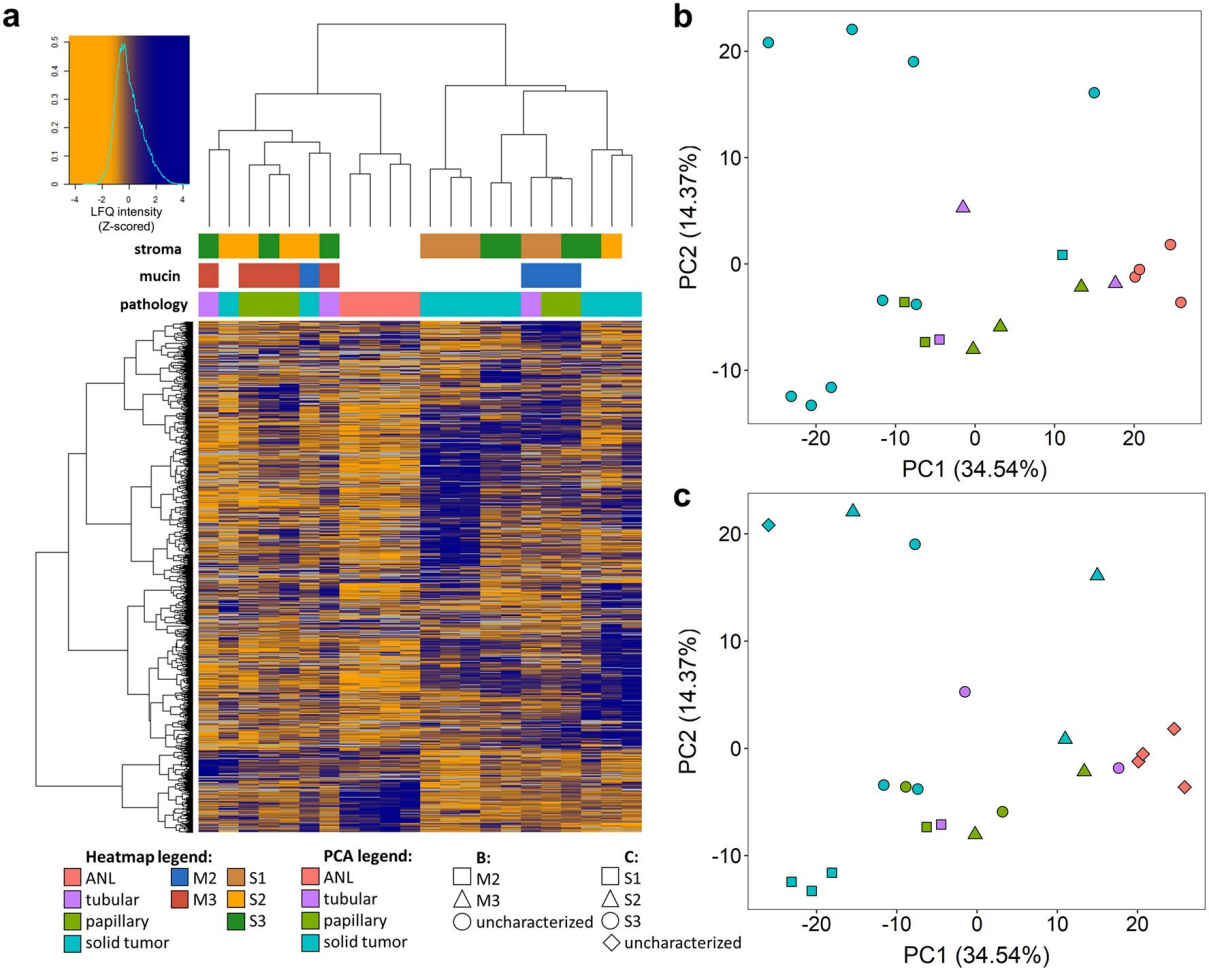
Heatmap created after hierarchical clustering, generated for proteins quantified in at least 10 regions (**a**) and principal component analysis for proteins quantified in all samples labelled by morphological classification and mucin content (**b**), morphological classification and stromal content (**c**). Figure was created in R 3.6.1 using RStudio 1.2.5001.

Hierarchical clustering was performed on proteins quantified in at least 10 regions and a heatmap was generated (Fig. 8a). Principal component analysis (PCA) was performed on proteins quantified in all the samples (Fig. 8b,c). Adjacent normal regions clustered together based on proteomic profiles and were well separated from the tumor regions in the PCA plot (Fig. 8b,c). Of the solid tumor regions, 8 out of the 10 also clustered together, while papillary regions were divided into two groups based on their mucin content (M2 vs M3). The three tubular regions did not cluster together in hierarchical clustering and were far apart in PCA.

In both the hierarchical clustering and PCA, it can be observed that M2 and M3 regions are well distinguishable, with only one M2 region falling within the M3 regions (Fig. 8a,b). However, this outlier was a solid region, in contrast to the other ones, which were either tubular or papillary. As for stromal content, S1 regions create two subgroups in clustering and PCA, containing 3 and 2 samples (Fig. 8a,c). 3–3 samples from S2 and S3 regions were also located close in the PCA plot, but the other samples showed large differences and did not group together.

**Figure 9:**
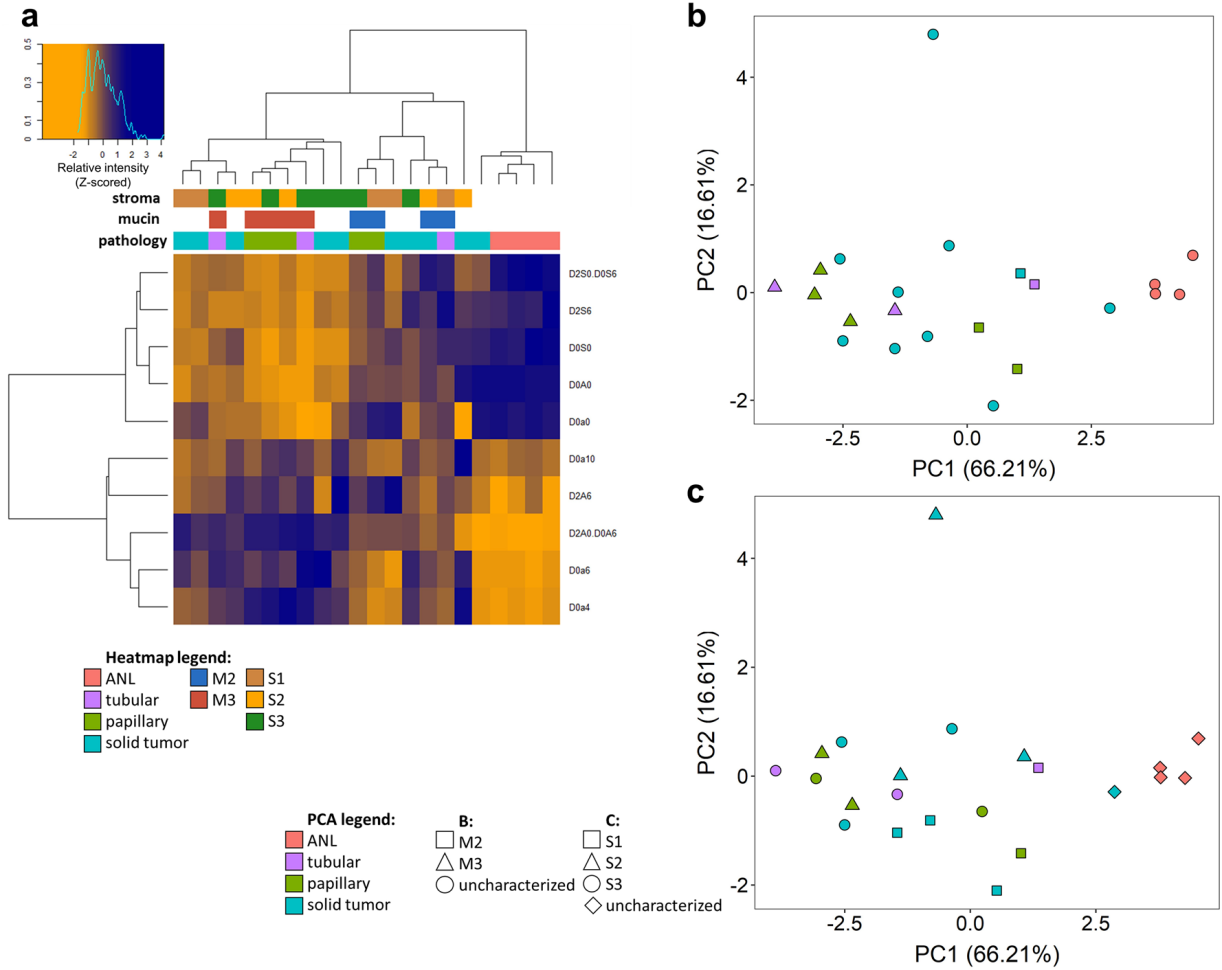
Heatmap created after hierarchical clustering, generated for CS and HS disaccharides (**a**) and principal component analysis for these components labelled by morphological classification and mucin content (**b**), morphological classification and stromal content (**c**). Figure was created in R 3.6.1 using RStudio 1.2.5001.

In GAG-omic analysis, the disaccharides clustered together in hierarchical clustering according to their sulfation position (Fig. 9a). All the *O*-sulfated disaccharides (D0a4, D0a6 and D0a10 CS disaccharides, and D2A6 and D2A0 + D0A6 HS disaccharides) belonged to one of the two main component clusters, while the other cluster included the *N*-sulfated HS (D2S0 + D0S6, D2S6, D0S0) and the non-sulfated disaccharides (D0A0, D0a0). *O*-sulfated components were typically overexpressed in tumor regions compared to adjacent normal ones, while *N*-sulfated and non-sulfated components were more abundant in adjacent normal regions.

In the hierarchical clustering and PCA, the adjacent normal regions were almost completely separated (Fig. 9). As in proteomics, papillary tumor regions appeared in two subgroups with different mucin content: members of one belonged to M2, while members of others to M3 sample group. Tubular and solid regions showed different characteristics and did not cluster together. Based on the mucin content, M2 and M3 regions were well separated in PCA and formed perfectly distinct clusters in hierarchical clustering (Fig. 9a,b), which was also fulfilled for CS and HS separately (see Supplementary Fig. S5). In contrast, the S1, S2 and S3 domains in the PCA overlapped to a considerable extent (Fig. 9c) and showed poor separation in hierarchical clustering (Fig. 9a).

Based on our hierarchical clustering and PCA results, it can be concluded that the relation between mucin and both protein and GAG content of the tissue region is especially strong, while stromal content has a minor effect on these profiles.

## Discussion

In this study, we performed proteomic and GAG-omic analysis on 22 tissue regions from 7 ALK rearranged lung cancer patients, grouped according to different histopathological aspects (morphology, mucin and stroma content).

In our proteomic studies, we observed 24 and 29 proteins involved in translation and RNA splicing being differentially expressed between papillary and solid morphological tumor types, e.g. ribosomal protein S28 and small nuclear ribonucleoprotein, respectively. Several RNA-binding proteins, such as eukaryotic translation initiation factor 3 subunit A and polyadenylate-binding protein 1, showed significant difference in other tumor comparisons as well, implying that protein synthesis may differ considerably in all three tumor morphological groups. RNA binding proteins play a pivotal role in maintaining homeostasis of gene expression by controlling RNA splicing, polyadenylation, mRNA localization, mRNA stability and translation^30^. A known phenomenon in the literature is that RNA binding proteins are aberrantly expressed in cancer, thereby affecting the expression and function of oncogenes and tumor suppressor genes^31^.

Our results showed that proteins being differentially expressed between adjacent normal and individual morphological tumor regions were associated not only with protein synthesis, but also with a number of biological processes or localizations, e.g. ECM organization (37 proteins in normal-solid comparison), oxidation–reduction processes (61 proteins in normal-papillary comparison), protein folding (33 proteins in normal-solid comparison), and many differentially expressed proteins were located in the mitochondria (69 proteins in normal-solid comparison).

The fact that 23–37 differentially expressed proteins (e.g. collagen alpha-1(IV) chain, laminin subunit alpha-3, basement membrane-specific HSPG core protein, etc.) are associated with ECM organization, is consistent with former observations that the highly dynamic balance between ECM synthesis and secretion is perturbed in cancer^32^. The main components of ECM are fibrous proteins (collagen, laminin, elastin) and PGs, which create a unique, tissue-specific composition^33^. If the organization of ECM is dysregulated, this composition is altered and is unable to fulfill its function, which can lead to cancer development or progression^34^. We observed the lower expression of these proteins in all tumor regions compared to adjacent normal ones, which is consistent with previous findings in the literature for NSCLC^23^.

Proteins taking part in oxidation–reduction processes (e.g. glucose-6-phosphate isomerase, catalase), protein folding (e.g. 78 kDa glucose-regulated protein, DnaJ heat shock protein family member b1) and proteins in mitochondria (e.g. ATP synthase subunit beta, prohibitin-2) were found to be mostly overexpressed in tumors relative to adjacent normal regions. Proteins discussed here as part of oxidation–reduction processes are mostly involved in metabolic processes, such as carbon and carbohydrate metabolism. Cancer cells show an altered metabolism, characterized by reduced oxidative phosphorylation and increased aerobic glycolysis, which enables them to meet the increased energy and biomass demand of the cell^35^. The maintenance of protein homeostasis in the endoplasmic reticulum (ER) is mediated by the unfolded protein response. Impaired protein folding or increased protein secretion can trigger the accumulation of unfolded or misfolded proteins in the ER, which has been implicated in cancer, among other diseases^36^. Tumor cells upset the balance of mitochondrial dynamics as well, thus an increased mitochondrial fission has already been observed in a variety of cancer cells, including lung cancer^37^.

In our GAG-omic analysis, tumor regions showed an increased expression level and sulfation of CS and HS chains compared to adjacent normal regions, while smaller differences could be discovered between individual tumor morphologies. The three types of tumor regions showed the largest difference in the total amount of HS. Changes in the sulfation pattern of GAGs have an important role in governing extracellular signaling^38^ and thereby regulating tumor invasion, proliferation, angiogenesis and metastasis^39,40^. Differences in the sulfation pattern and in the rate of sulfation have already been described in several types of cancer (e.g. breast, prostate, ovarian cancer)^41–44^. Interestingly, we found an increase in the rate of HS sulfation in tumors, in opposition with recent cancer literature examples, where decreased HS sulfation has been reported^45,46^. This finding calls for future studies investigating larger number of ALK rearranged lung tumors.

We observed a 1.1–1.5-fold increase in the 6S/4S CS disaccharide ratio in tumors with different morphologies compared to the adjacent normal regions. It is known that low-grade chronic inflammation occurs during tumor development, and 6-*O*-sulfation in CS chains has an anti-inflammatory effect^47^, which can explain the increase in the 6S/4S ratio noted in our experiments.

We also observed a considerable decrease in the *N*-sulfation *vs O*-sulfation ratio in tumor regions compared to the adjacent normal ones (total *N*/*O* sulfate contribution decreased by 4.4–9.4). Some studies indicate altered expression of NDST genes—coding for *N*-deacetylase/*N*-sulfotransferase enzymes—in cancer^48,49^, which may result in altered *N*/*O*-sulfation ratio.

Differentially expressed PG core proteins between morphological types were agrin, perlecan and collagen alpha-1(VIII) chain, all of which are basement membrane PGs and play an important role in cancer growth and angiogenesis^50^.

By comparing the proteomic results of M2 and M3 tumor regions, it could be observed that differentially expressed proteins were connected to some of the previously discussed processes (e.g. translation in protein expression, ECM organization and protein folding) that play a vital role in cancer development and progression. Nevertheless, it should be emphasized that tumor regions containing medium (M2) and high (M3) amounts of mucin differ greatly in both protein and GAG content, indicating that the proteomic and GAG-omic profiles are influenced by mucin content.

We observed a considerable increase in the CS and HS content in M3 regions, accompanied by the alteration of sulfation patterns. The synthesis of mucin-type *O*-glycans is catalyzed by GALNT enzymes^51^. Different expression of GALNT and GCNT genes coding for GalNAc and GlcNAc transferase enzymes has already been described in several types of cancer, including lung cancer^52,53^. It is hypothesized that—similarly to these enzymes—glycosyltransferases involved in CS and HS biosynthesis may also be upregulated.

Small leucine rich proteoglycans, e.g. decorin, biglycan, lumican and fibromodulin were all overexpressed in M3 regions relative to M2 ones. The overexpression of these PGs has been observed in various types of cancer and they can regulate cancer cell multiplication, angiogenesis and migration^54^. Increased amount of versican PG was also observed, which plays an important role in proliferation, migration and angiogenesis, and high amount of versican is usually accompanied by poor patient outcome^55^.

The proteomic changes between S1, S2 and S3 regions were associated with processes discussed earlier, such as RNA binding, ECM organization and oxidation–reduction processes. We observed that the CS content increased from S1 to S2 group by 1.7 and from S2 to S3 by 1.7, while the same ratios for the total HS amount were 1.6 and 2.2, respectively. Since PGs are important components of the ECM, the elevated amount of ECM might explain that tissues with higher stromal content contain more CS and HS chains. Five PGs (decorin, biglycan, lumican, versican and perlecan) were differentially expressed between S1 and S3 regions, which are involved in biological processes discussed earlier. However, based on hierarchical clustering and PCA, stromal content has low effect on the proteomic and GAG-omic profile of the tumor.

## Conclusions

Proteomic and GAG-omic hierarchical clustering and PCA showed that both profiles were strongly related to mucin content and partially related to morphological classification, while the profiles were less dependent on the stromal content of the tissue regions.

Our proteomic study revealed that differentially expressed proteins between different morphological tumor regions were mostly involved in translation and RNA splicing, while those between adjacent normal and tumor regions were involved in other biological processes as well, e.g. extracellular matrix organization, oxidation–reduction process and protein folding. Similar processes proved to be dysregulated between M2 and M3 as well as between S1, S2 and S3 sample groups. Significant difference was observed in the amount of several PG core proteins in all comparisons.

GAG disaccharide analysis demonstrated that the total and relative amount of CS and HS disaccharides depended greatly on the morphological type and mucin content of the tissue, while stromal content is mainly related to the total amount of CS and HS. The observed differences (e.g. 6S/4S and *N*/*O*-sulfation ratio) allowed us to infer biological processes involved in the disease, which are mostly consistent with previous observations in the literature.

Overall, our pilot study provides an excellent starting point for further studies investigating larger number of ALK rearranged lung cancer patients, as well as for proteomic and GAG-omic characterization of other gene rearrangements causing adenocarcinoma.

## Methods

### Selection of samples

22 tissue regions from 7 patients have been investigated. Tumor regions were classified based on morphological classification, mucin and stromal content. The morphological classification’s groups were the following: solid tumor (*n* = 10), tubular tumor (*n* = 3), papillary tumor (*n* = 5), adjacent normal (*n* = 4). The stroma and mucin content of tumor regions were scored from one to three. Mucin scores of 1, 2, 3 indicate 1–33%, 33–66% and 66–100% mucin and mucin producing tumor of total area, respectively. Stroma scores of 1, 2, 3 indicate 1–33%, 33–66% and 66–100% neostroma of total area, respectively. The regions were analyzed by board certified pathologists, they evaluated the slides via semiquantitative assessment as performed in routine histopathological diagnostics. The properties of the individual regions are collected in Table 1, while the pictures of the tissue sections and regions are illustrated in Supplementary Fig. S6. The study was conducted in accordance with the Declaration of Helsinki, and approved by the Institutional Review Board of Ethics Committee of Ministry of Health, Hungary (TUKEB permit numbers: 2521-0/2010-1018EKU, 52614-4-213EKU). Informed consent was obtained from all participants.

### Materials

Heparin lyase I, II, III, CS and HS disaccharide standards were purchased from Iduron (Manchester, UK), Trypsin Gold and Trypsin/Lys-C mixture from Promega (Madison, WI, USA), LC–MS grade solvents (water, acetonitrile (ACN), xylene, ethanol, methanol (MeOH), formic acid (FA)) from VWR (Debrecen, Hungary), Rapigest from Waters (Budapest, Hungary). Acetic acid, Chondroitinase-ABC, ammonium bicarbonate (ABC), ammonium formate, ammonium acetate, sodium citrate, citric acid, dithiothreitol (DTT), glycerol, iodoacetamide (IAA), trifluoroacetic acid (TFA), heptafluorobutyric acid (HFBA), Tris, NH_3_ and Ca(OH)_2_ were obtained from Merck (Darmstadt, Germany). Pierce C_18_ and graphite SPE tips were purchased from Thermo Fischer Scientific (Unicam, Budapest, Hungary).

### Preparation of tissue sections

From the formalin-fixed, paraffin-embedded (FFPE) tissues 6 µm thick sections were mounted on SuperFrost Ultra Plus microscopic slides. First, tissue sections were baked at 60 °C for 2 h. Next, dewaxing was carried out by incubating the tissues subsequently with xylene (2 × 5 min), ethanol (2 × 3 min), ethanol–water mixtures (90% ethanol: 3 min, 70% ethanol: 3 min), 10 mM ABC solution (5 min) and water (1 min). Next, antigen retrieval was carried out by boiling the tissue sections at 80–85 °C in citrate buffer (94.6 mM sodium citrate, 20.8 mM citric acid, pH 6) for 30 min.

### Proteomics workflow

#### On-tissue digestion

A serial on-tissue digestion was carried out. First, 2 μL of 0.1% RapiGest + 5 mM DTT + 20% glycerol was pipetted on the regions and incubated in a humidified box at 55 °C for 20 min. Second, 2 μL of 25 mM ABC + 10 mM IAA + 20% glycerol solution was added to the drops and incubated at room temperature in the dark for 20 min. The digestion was first performed in two cycles with 2–2 μL Lys-C/trypsin mixture (40 ng/μL in a mixture of 50 mM ABC and 15% glycerol), then in 3 cycles with 2–2 μL trypsin solution (200 ng/μL in a mixture of 50 mM ABC and 15% glycerin). During each cycle, the samples were incubated in a humidified box at 37 °C for 40 min^56^. Finally, the liquid was pipetted from the tissue surface and the peptides remaining on the surface were extracted with 5 × 2 μL of 10% acetic acid solution by repeated pipetting. The samples were dried down and stored at − 20 °C until the purification.

#### Solid phase extraction purification

Sample purification was performed on a reversed-phase C_18_ spin cartridge, as previously published^57,58^. Briefly, the cartridge was activated with 2 × 200 µL 50% MeOH and 2 × 200 µL 0.5% TFA + 5% ACN, then equilibrated with 2 × 200 µL of 0.1% HFBA solution. Samples were applied and reapplied once in 50 µL 0.1% HFBA solution. The tip was washed with 2 × 100 µL of 0.1% HFBA, and peptides were eluted with 2 × 50 µL of 0.1% TFA + 70% ACN solution and 50 µL of 0.1% FA + 70% ACN solution after 1 min incubation. All the used materials, except elution solvent were thermostated at 4 °C. After each step, the samples were centrifuged at 2000 rpm for 1 min. The solvents were evaporated and the samples were stored at − 20 °C until further use.

#### nanoUHPLC-MS/MS measurements

Samples were dissolved in 8 μL 0.1% FA + 2% ACN, of which 6 μL was injected. Measurements were carried out on a Bruker Maxis II Q-TOF mass spectrometer (Bruker, Bremen, Germany) coupled to a Dionex Ultimate 3000 RSLC nanoUHPLC (Thermo Fischer Scientific, Waltham, MA). Trapping was performed on a Thermo Fischer Acclaim PepMap100 C_18_ column (100 μm × 20 mm), followed by the separation of peptides on an Acquity M-Class BEH130 C_18_ analytical capillary column (1.7 μm, 75 μm × 250 mm, Waters, Milford, MA). The flow rate was 0.3 μL/min, while solvents A and B were 0.1% FA in water and in ACN, respectively. The applied gradient program was as follows: solvent B content was increased from 4 to 25% in 75 min, then to 40% in 15 min and 90% in 1 min, washed for 5 min, and finally the column was equilibrated with 4% B for 20 min. Examples of proteomic base peak chromatograms are shown in Supplementary Fig. S7.

The MS ion source was a CaptiveSpray nanoBooster used in positive mode, with a capillary voltage of 1150 V, a gas pressure of 0.2 bar and a drying gas flow rate of 3 L/min. MS and MS/MS spectra were recorded in the mass range of 150–2200 *m*/*z* with a cycle time of 2.5 s. MS spectra were recorded at a frequency of 3 Hz, while MS/MS spectra were taken at 16 Hz or 4 Hz, depending on the intensity of the precursor ions. The collision energy was determined by the control software based on the *m*/*z* value and charge of the precursor ion. For mass calibration, sodium formate was used and data was recalibrated by the Compass DataAnalysis software 4.3 (Bruker Daltonics, Bremen, Germany).

#### Data analysis and visualization

Byonic^59^ software was used for the identification of proteins, using the Swiss-Prot human database (access date: 24.11.2020), a mass accuracy of 5 ppm for precursor ions and 10 ppm for fragment ions was set. Carbamidomethylation of cysteine amino acids was set as a fixed modification, while deamidation of asparagine and glutamine, and oxidation of methionine were set as variable modifications. A maximum of 2 missed cleavage sites were allowed. Proteins with at least 2 reliably identified unique peptides (LogProb greater than 1.3) and a LogProb greater than 2 were accepted. For label-free quantification of proteins, MaxQuant^60^ 1.6.17.0 software was used with the settings shown in Supplementary Table S4.

Statistical evaluation and visualization of the results were performed in R 3.6.1^61^ using RStudio 1.2.5001^62^. Plots were made with ggplot2 and gplot packages. Proteins quantified in at least 3-3 samples in at least 2 sample groups were considered for further analysis. Protein expression levels between sample groups were compared using Wilcoxon signed-rank test, and nominal *p* values less than 0.05 were considered significant, except for the comparison of tubular tumor and adjacent normal regions where 0.057 was used as this is the smallest possible *p* value when comparing two groups with 3 and 4 elements. For hierarchical clustering, proteins quantified in at least 10 regions and for PCA, proteins quantified in all regions were used. Hierarchical clustering was carried out using the heatmap.2 function with Ward’s clustering method “ward.D2” from the hclust function, while PCA was performed using the prcomp function, with variable scaling and default settings.

Mapping of protein interactions was performed using the STRING webserver^63^. The minimum required interaction score was set to high confidence (0.700), the meaning of network edges to confidence, disconnected nodes were hidden in the network and the default was used for the other settings.

### GAG-omics workflow

#### On-tissue digestion of chondroitin sulfate

First, CS digestion was performed by using 5 × 2 μL of digestion solution (2 mU/μL chondroitinase-ABC enzyme in a solution of 20 mM Tris (pH 7.6) + 2.5 mM ammonium acetate + 10% glycerol) on each region. Samples were incubated in a humidified box at 37 °C for 60 min during each cycle, and the last drop was left on the tissue surface for a total of 24 h from the start of digestion^56,64^. The liquid drops were pipetted off and the remaining disaccharides were extracted by 5 × 2 μL of 1% NH_3_ solution, then the drops were collected and the solvents were evaporated. The tissue surfaces were washed with 10 mM Tris (pH 7.6) solution.

#### On-tissue digestion of heparan sulfate

Following CS digestion, HS chains were digested on the same spots. The concentration of the enzyme solution applied was 0.5 mU/μL for heparin lyase I and 0.1 mU/μL for heparin lyase II and III, in a solution of 20 mM Tris (pH 7.6) + 2.5 mM Ca(OH)_2_ + 10% glycerol. The digestion was performed for 48 h in a humidified box at 37 °C with 5 × 2 μL digestion solution, which were added after 0, 1, 2, 19 and 26 h from the start of the digestion^56,64^. Extraction was carried out the same way as for CS disaccharides.

#### Cotton-HILIC + graphite SPE purification

For purifying the GAG disaccharides, a combined Cotton-HILIC + graphite SPE procedure was carried out, as described elsewhere^65^. In all steps, the samples were centrifuged at 2500 rpm for 1 min. The first part was done by using self-packed cotton-HILIC pipette tips. The stationary phase was activated with 50 µL of 60% ACN solution, then conditioning was performed with 2 × 50 µL of 1% TFA + 95% ACN solution. Samples were applied in 30 µL of 1% TFA + 95% ACN solution, and the flow-through was reapplied onto the tip twice. The tip was washed with 50 µL of 1% TFA + 95% ACN solution, and the flow-through from loading and wash were combined and dried down for further purification. The elution was performed with 2 × 10 µL of 1% and 5% NH_3_ solution heated to 40 °C for the purification of CS and HS disaccharides, respectively.

In the next step, the flow-throughs of loading and wash fractions were further purified on a Thermo Pierce graphite tip. 2 × 100 µL of 0.1% TFA + 80% ACN solution was used for activation, then 2 × 100 µL of water for conditioning. The samples were applied in 50 µL of water and centrifuged after 2 min of incubation. The flow-through was reapplied, then the tip was washed with 3 × 100 µL of water. Finally, 3 × 50 µL 0.05% TFA + 40% ACN solution was used for elution. The elution fractions from this and the previous step were combined, solvents were evaporated, and samples were stored at − 20 °C until further use.

#### HPLC–MS measurements

The samples were dissolved in 8 μL 10 mM ammonium formate + 75% ACN (pH 4.4) solution, of which 1.5 μL was injected. Measurements were performed by using a Waters Select Series cyclic ion mobility mass spectrometer (Milford, MA) coupled to a Waters Acquity I-class UPLC (Milford, MA). Disaccharides were separated on a self-packed HILIC-WAX capillary column (250 μm × 10 cm). The flow rate was 10 μL/min, while solvent A and B consisted of 10 mM and 65 mM ammonium formate + 75% ACN (pH 4.4) solution, respectively^66,67^. CS disaccharides were separated by isocratic elution (8% B) for 10 min, in contrast to the HS disaccharides, which were eluted with the following gradient program: B content was elevated from 6 to 100% in 0.5 min, washing for 7.5 min, then decreasing back to 6% B in 0.5 min, and finally 16.5 min equilibration.

For mass spectrometric detection, a low-flow ESI source was used in negative mode, the capillary voltage being 1.9 kV, the cone voltage 20 eV, and the temperature of the ion source 120 °C. When measuring HS disaccharides, only MS1 spectra were recorded, the trap collision energy was set to 6 eV, and the transfer collision energy to 3 eV. For the measurements of CS disaccharides, MS1 and MS/MS spectra were taken, to distinguish the isomer pair D0a4 and D0a6. For these, fragmentation occurred in the transfer with 32 eV collision energy. MS spectra were taken in the mass range of 180–680 and 200–600 *m*/*z* for HS and CS samples, respectively. As an example, extracted ion chromatograms of characteristic ions for CS and HS disaccharides are shown in Supplementary Figs. S8 and S9.

#### Data analysis and visualization

Chromatographic peaks were integrated using TargetLynx incorporated into the MassLynx V4.2 software. Data curation was carried out in Microsoft Excel. The results were plotted using OriginPro 2018. For the GAG-omics data, Wilcoxon signed-rank test was used for statistical evaluation in the same way as for the proteomics data. Statistical evaluation, principal component analysis and hierarchical clustering were carried out in R 3.6.1^61^ using RStudio 1.2.5001^62^, similarly to proteomics.

## Supplementary Information

Supplementary Information 1.

Supplementary Information 2.

Supplementary Information 3.

## Data Availability

The data of the proteomics measurements are available in the MassIVE repository under the https://doi.org/doi:10.25345/C5319S63V link and can be downloaded via FTP (ftp://massive.ucsd.edu/MSV000089286/). The GAG-omics data presented in this study have been deposited in the GlycoPOST database^69^ under the accession number of GPST000303.
