# Supplementary material for: Inter- and intratumoral proteomics and glycosaminoglycan characterization of ALK rearranged lung adenocarcinoma tissues: a pilot study
Source: Sci Rep. 2023 Apr 17;13:6268. doi: 10.1038/s41598-023-33435-1 (PMC10110559; doi:10.1038/s41598-023-33435-1)
Supplement: Supplementary file 3 — Supplementary Information 3. [file 41598_2023_33435_MOESM3_ESM.pdf]

Supplementary material to the paper

Inter- and intratumoral proteomics and glycosaminoglycan characterization of ALK rearranged lung adenocarcinoma tissues – a pilot study

Mirjam Balbisi[a,b], Simon Sugár[a,b], Gitta Schlosser[c], Beáta Szeitz[d], János Fillinger[e], Judit Moldvay[f], László Drahos[a], A.Marcell Szász[d], Gábor Tóth[a,], Lilla Turiák[a,b,*]*

[a]MS Proteomics Research Group, Research Centre for Natural Sciences, Magyar tudósok körútja 2., H-1117 Budapest, Hungary

[b]Ph.D. School of Pharmaceutical Sciences, Semmelweis University, Üllői út 26, H-1085, Budapest, Hungary

[c]MTA-ELTE Lendület Ion Mobility Mass Spectrometry Research Group, Eötvös Loránd University, Pázmány Péter sétány 1, H-1117, Budapest, Hungary

[d]Division of Oncology, Department of Internal Medicine and Oncology, Semmelweis University, Budapest, Hungary

[e]Department of Pathology, National Korányi Institute of Pulmonology, Budapest, Hungary

[f]1st Department of Pulmonology, National Korányi Institute of Pulmonology, Budapest, Hungary

***Corresponding authors**

Lilla Turiák
Research Centre for Natural Sciences
Magyar tudósok körútja 2., H-1117, Budapest, Hungary
Email: turiak.lilla@ttk.hu
Tel: +36 1 382 6548

Gábor Tóth
Research Centre for Natural Sciences
Magyar tudósok körútja 2., H-1117, Budapest, Hungary
Email: toth.gabor@ttk.hu
Tel: +36 1 382 6542

Table of contents

Table S1. Statistical results observed for CS and HS disaccharides for all the comparisons performed (presented as a separate xlsx file).

Table S2. List of differentially expressed proteins in each comparison according to morphological classification (presented as a separate xlsx file).

Table S3. Counts in network, strengths and false discovery rates for the examined biological processes and localizations marked on protein networks (Figures 2, 5, S1, S2).

Figure S1. Protein interaction networks built on differentially expressed proteins between **a:** adjacent normal and tubular, **b:** adjacent normal and papillary tumor regions.

Figure S2. a: Differentially expressed proteins; **b:** Protein interaction networks built on differentially expressed proteins between two sample groups, **c:** Differentially expressed proteoglycan core proteins between regions with different stromal content. (*: $p<0.05$, **: $p<0.01$)

Figure S3. a: Relative amount of CS disaccharides (%), **b:** total amount of CS disaccharides (pmol), **c:** average rate of CS sulfation, **d:** 6S/4S ratio for regions with different stromal content. Error bars represent standard deviation.

Figure S4. a: Relative and total amount of HS disaccharides, **b:** sulfation characteristics for regions with different stromal content. Error bars represent standard deviation.

Figure S5. Heatmap created after hierarchical clustering, generated for **a:** CS disaccharides, and **b:** HS disaccharides. Figure was created in R 3.6.1 using RStudio 1.2.5001.

Figure S6. H&E stained images of the examined tissue sections and numbering of the regions.

Figure S7. Base peak chromatograms in proteomic measurements from regions with different morphological classifications. Figure was created in Compass DataAnalysis 4.3.

Table S4. Settings used for quantification of proteins in MaxQuant.

Figure S8. Extracted ion chromatograms of CS disaccharide measurements from regions with different morphological classifications. Figure was created in MassLynx V4.2.

Figure S9. Extracted ion chromatograms of HS disaccharide measurements from regions with different morphological classifications. Figure was created in MassLynx V4.2.

Table S3. Counts in network, strengths and false discovery rates for the examined biological processes and localizations marked on protein networks (Figures 2, 5, S1, S2).

	Process / Localization	Count in network	Strength	False discovery rate
**Papillary – solid**	RNA splicing	29 of 396	0.88	8.94E-14
	Translation	24 of 366	0.83	2.12E-10
**Normal – solid**	Extracellular matrix organization	37 of 338	0.62	2.56E-10
	Mitochondrion	69 of 1611	0.21	9.80E-04
	Protein folding	33 of 213	0.77	7.86E-13
	RNA splicing	48 of 396	0.66	4.46E-15
	Translation	81 of 366	0.92	3.07E-42
**Normal – tubular**	Extracellular matrix organization	23 of 338	0.74	3.16E-08
	Oxidation-reduction process	46 of 939	0.60	3.08E-12
	RNA binding	61 of 1649	0.48	5.80E-12
**Normal – papillary**	Extracellular matrix organization	26 of 338	0.62	2.97E-07
	Oxidation-reduction process	61 of 939	0.55	1.76E-14
	RNA binding	103 of 1649	0.53	9.09E-25
**M2-M3**	Extracellular matrix organization	18 of 338	0.82	1.93E-07
	Protein folding	9 of 213	0.72	0.0069
	Translation	13 of 366	0.65	0.0015
**S1-S2**	Extracellular matrix organization	29 of 338	0.85	4.08E-13
	Oxidation-reduction process	34 of 939	0.48	2.37E-06
	RNA binding	84 of 1649	0.63	2.71E-27
**S1-S3**	Extracellular matrix organization	30 of 338	0.86	1.77E-12
	Oxidation-reduction process	45 of 939	0.59	2.65E-11
	RNA binding	68 of 1649	0.53	4.23E-16

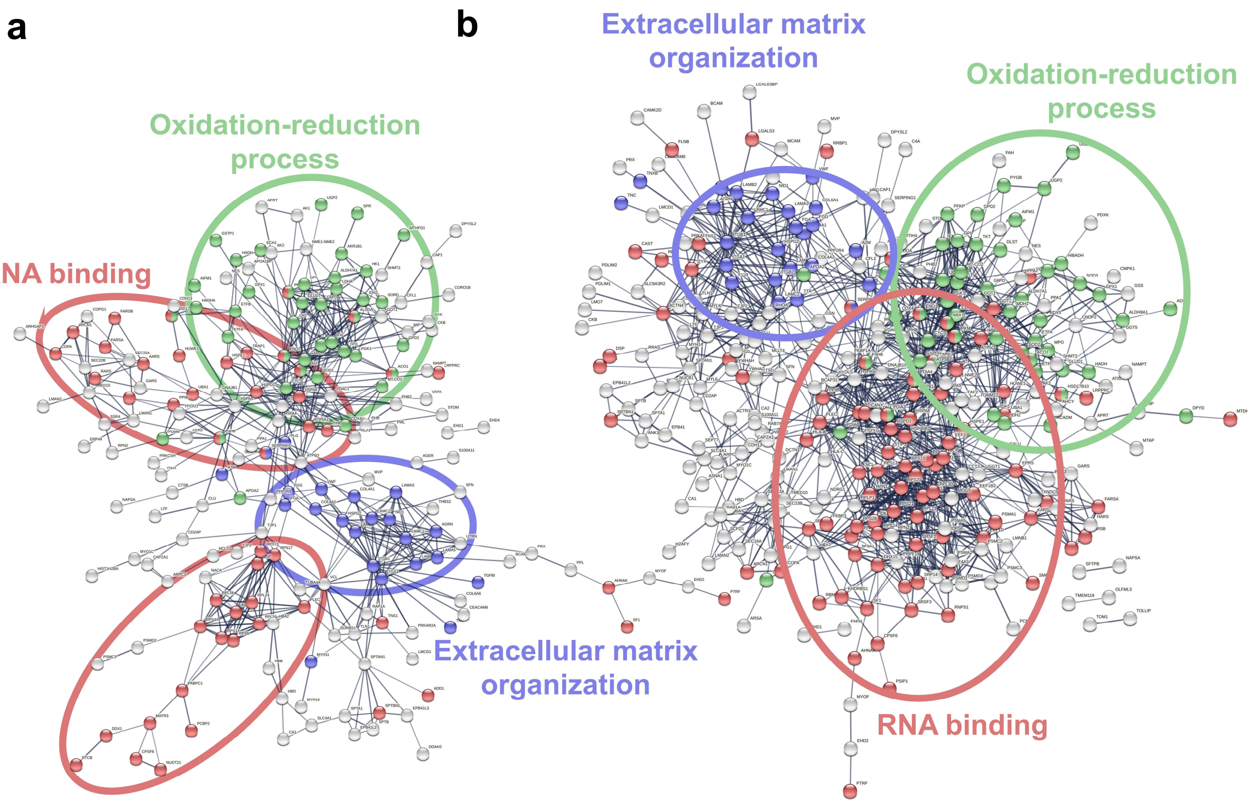

Figure S1. Protein interaction networks built on differentially expressed proteins between **a:** adjacent normal and tubular, **b:** adjacent normal and papillary tumor regions.

Comparison of regions with different stromal content

Regions containing low (S1), medium (S2) and high (S3) amounts of stroma are indicated by S1, S2 and S3, respectively. 248 proteins were differentially expressed between S1 and S2, 252 between S1 and S3, and 73 between S2 and S3 regions. For example, nidogen-1 and gamma-aducin were overexpressed, while phosphoglycerate kinase 1 and RNA-binding protein 25 were overerexpressed in S2 and S3 regions compared to S1 ones (Fig. S2/a). As for the differentially expressed proteins between S1 and S3 and between S2 and S3 regions, some of them were RNA binding proteins (84 and 68 of 1649), while others were involved in ECM organization (29 and 30 of 338) and oxidation-reduction processes (34 and 45 of 939) (Fig. S2/b). Members of clusters of RNA binding proteins and oxidation-reduction processes were mostly underexpressed in S2 and S3 tumor regions relative to S1 ones, while members of the ECM organization clusters were mostly overexpressed.

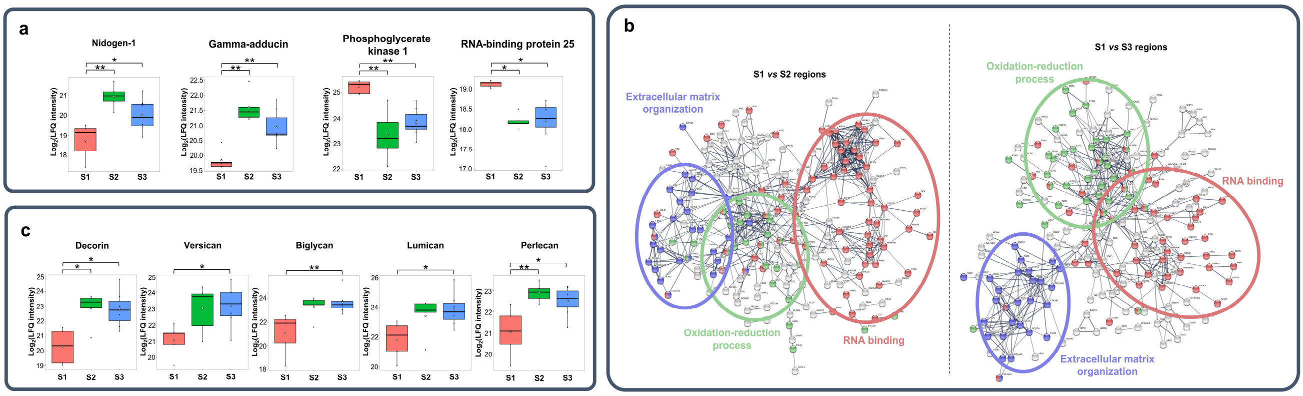

Figure S2. a: Differentially expressed proteins; **b:** Protein interaction networks built on differentially expressed proteins between two sample groups, **c:** Differentially expressed proteoglycan core proteins between regions with different stromal content. (*: $p<0.05$, **: $p<0.01$)

A significant overexpression of decorin (CSPG, FC=5.9) and perlecan (HSPG, FC=3.7) was observed in S2 regions compared to S1 ones, while S3 regions were more abundant in decorin (FC=5.8), versican (CSPG, FC=4.3), biglycan (CSPG, FC=6.2), lumican (KSPG, FC=4.1) and perlecan (FC=2.8) than S1 regions (Fig. S2/c). However, no PG core protein was found to be differentially expressed between S2 and S3 regions.

In CS analysis, the ratio of the non-sulfated D0a0 component in S1 regions was 1.6 and 1.5-fold higher than in S2 and S3 sample groups, while all the sulfated components occurred in larger proportion in the S2 and S3 regions (Fig. S3/a). As the amount of stroma in the tissue increases, the total amount of CS also increases rapidly: CS was 1.7 and 3.0 times more

abundant in S2 and S3 regions than in S1 (Fig. S3/b). The average rate of CS sulfation in S2 and S3 regions was approximately twice as high as in S1 regions (Fig. S3/c), while the 6S/4S CS disaccharide ratio was found to be higher in S1 and S3 sample groups than in S2 (Fig. S3/d).

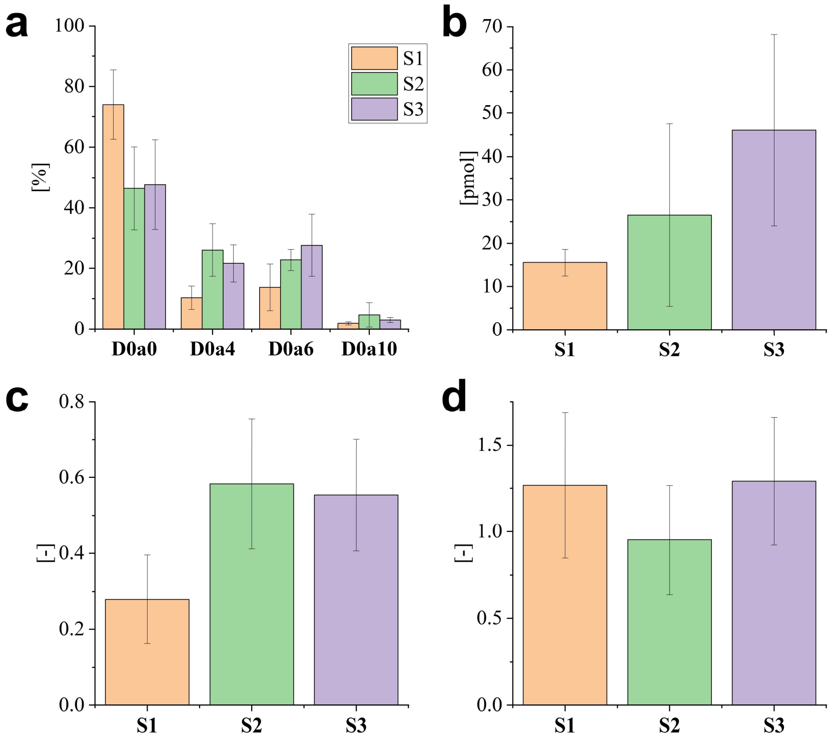

Figure S3. a: Relative amount of CS disaccharides (%), **b:** total amount of CS disaccharides (pmol), **c:** average rate of CS sulfation, **d:** 6S/4S ratio for regions with different stromal content. Error bars represent standard deviation.

In the case of HS, the three groups showed only slightly different sulfation pattern, but the total HS amount increased by a factor of 1.6 and 3.5 in S2 and S3 regions compared to S1 ones (Fig. S4/a). The average rates of HS sulfation were similar to each other, whereas the monosulfated, disulfated and total *N*/*O* ratios increased in S2 regions by 1.0-1.5 times, while the same quantities decreased by 1.2-1.7 times in S3 regions relative to S1 (Fig. S4/b).

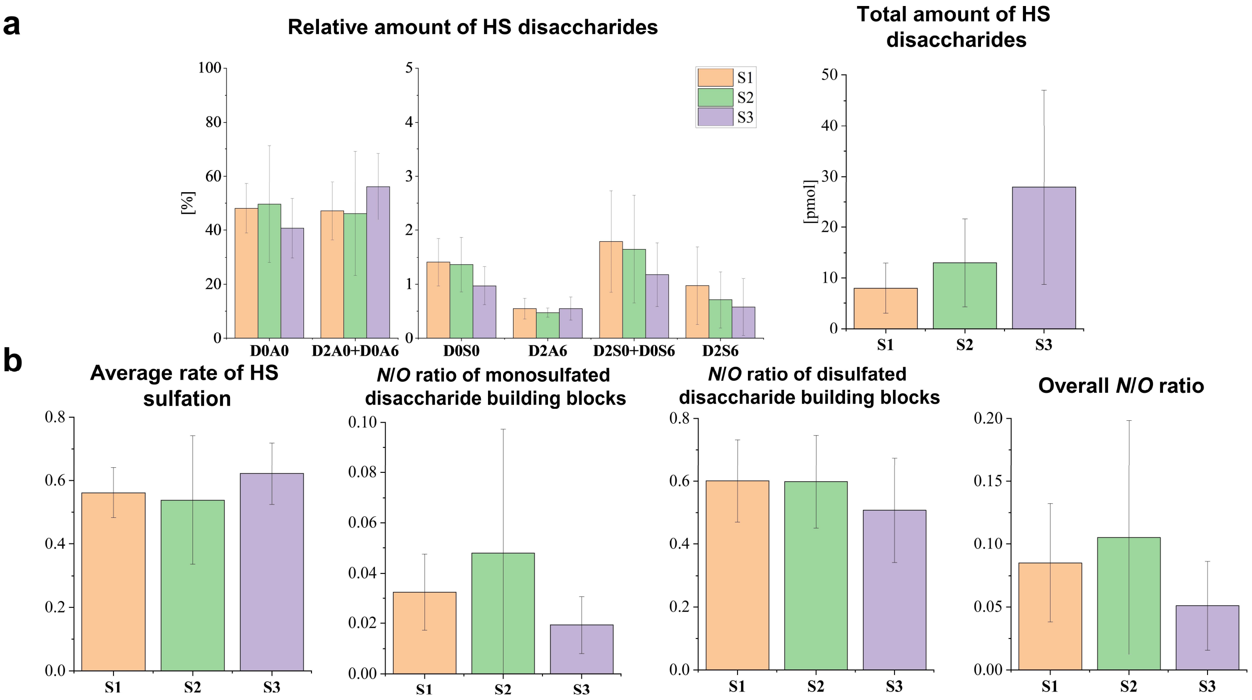

Figure S4. a: Relative and total amount of HS disaccharides, **b:** sulfation characteristics for regions with different stromal content. Error bars represent standard deviation.

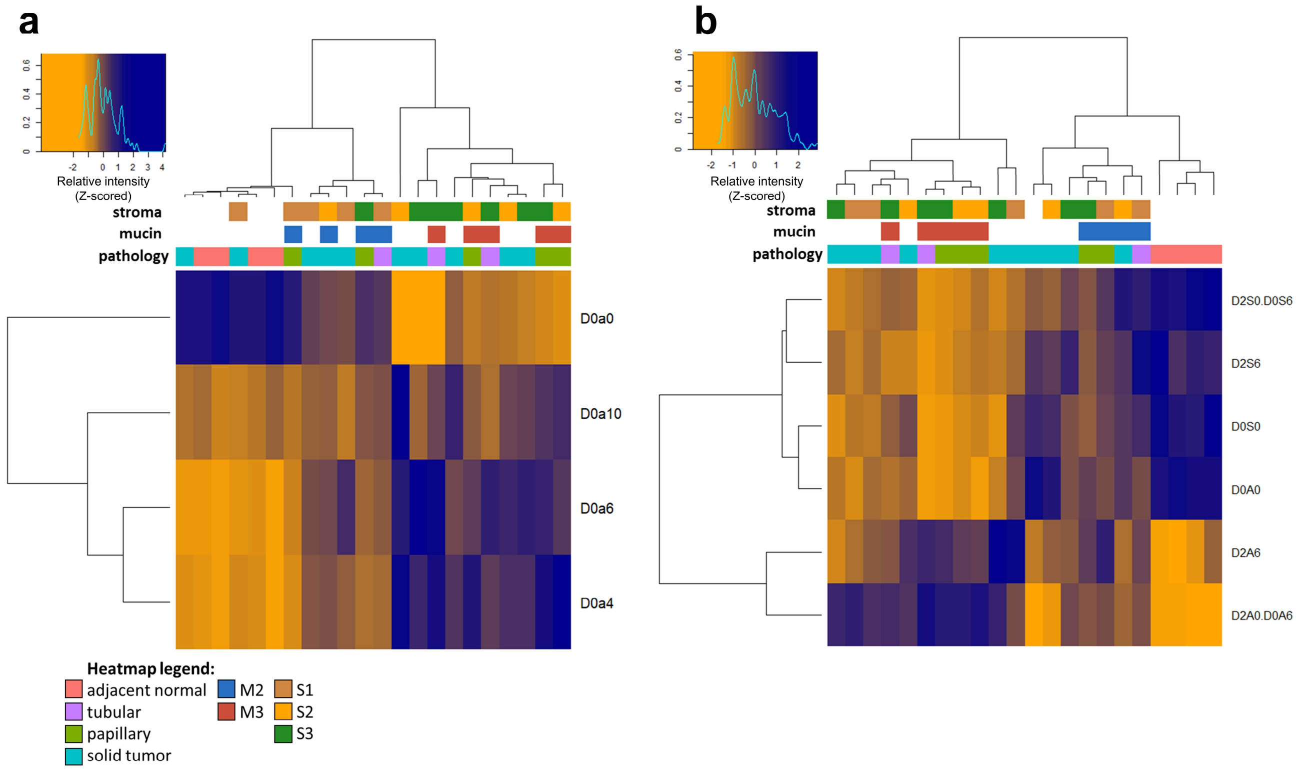

Figure S5. Heatmap created after hierarchical clustering, generated for **a:** CS disaccharides, and **b:** HS disaccharides. Figure was created in R 3.6.1 using RStudio 1.2.5001.

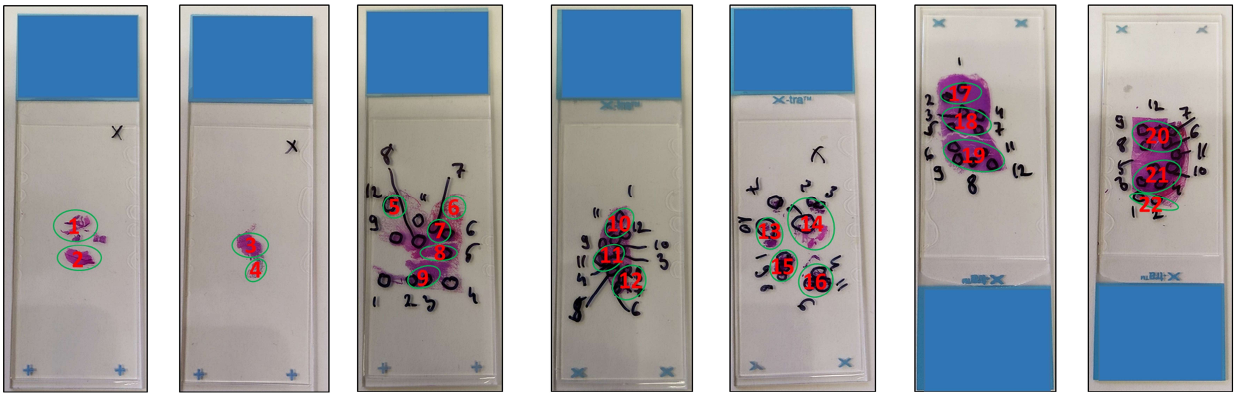

Figure S6. H&E stained images of the examined tissue sections and numbering of the regions.

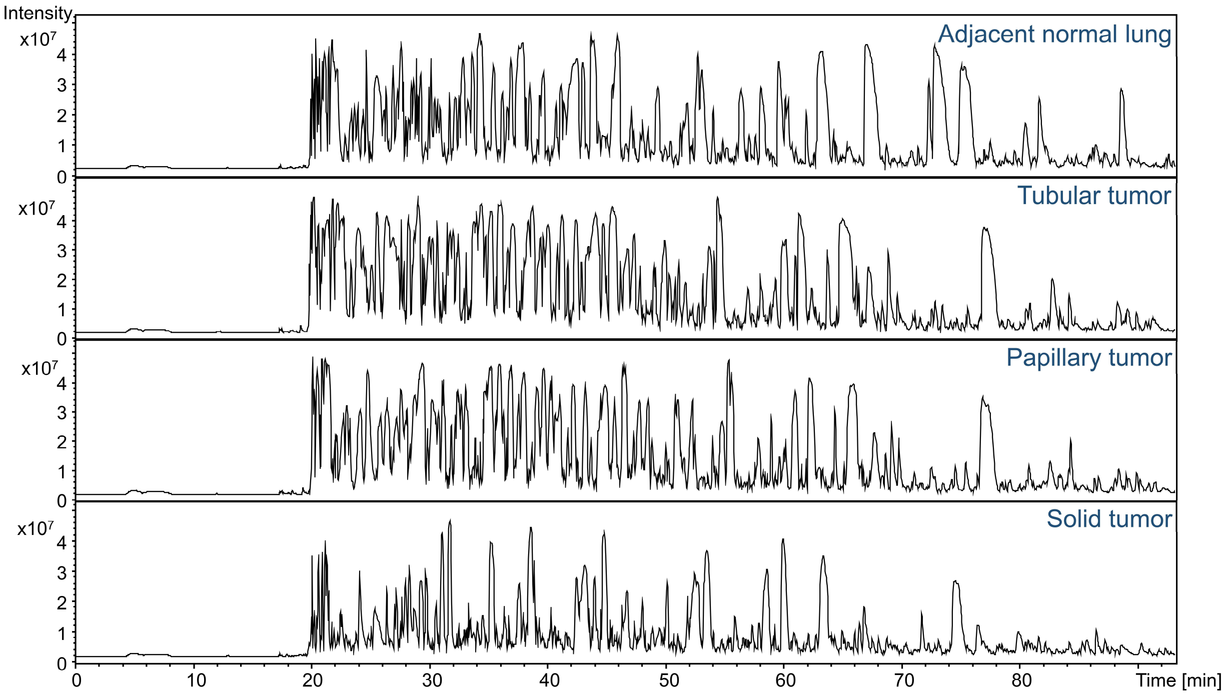

Figure S7. Base peak chromatograms in proteomic measurements from regions with different morphological classifications. Figure was created in Compass DataAnalysis 4.3.

Table S4. Settings used for quantification of proteins in MaxQuant.

Database	proteins identified by Byonic
Quantification	LFQ
LFQ min. ratio count	2
Instrument type	Bruker Q-TOF
First search peptide tolerance	20 ppm
Main search peptide tolerance	10 ppm
Max. charge	4
Intensity threshold MS1	30
Intensity threshold MS2	30
Enzyme	trypsin
Max. missed cleavage	2
Variable modifications	oxidation (M), deamidation (NQ)
Fixed modifications	carbamidomethyl (C)
Min. peptide length	7
Max. peptide mass	4600 Da
Identification	match between runs
Min. peptides	2
Min. unique peptides	1

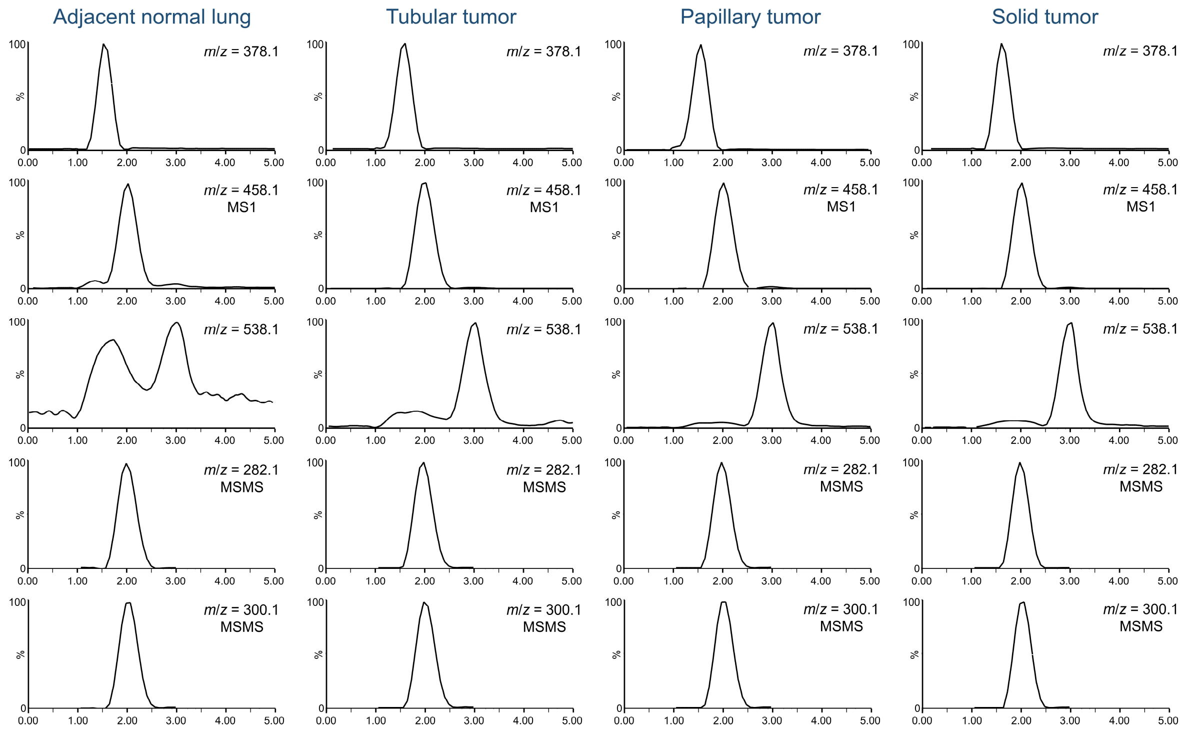

Figure S8. Extracted ion chromatograms of CS disaccharide measurements from regions with different morphological classifications. Figure was created in MassLynx V4.2.
The amount of D0a0 was determined by integrating the chromatographic peak areas corresponding to *m*/*z*=378.1, 400.1 and 416.1 at the retention time of 1.5 min, D0a4+D0a6 by integrating *m*/*z*=458.1, 480.1 and 496.1 at 2 min, and D0a10 by integrating *m*/*z*=538.1, 560.1 and 576.1 at 3 min. D0a6 and D0a4 disaccharides were quantified by determining the area ratio of the *m*/*z*=300.1 and 282.1 peaks, using a quadratic calibration function.

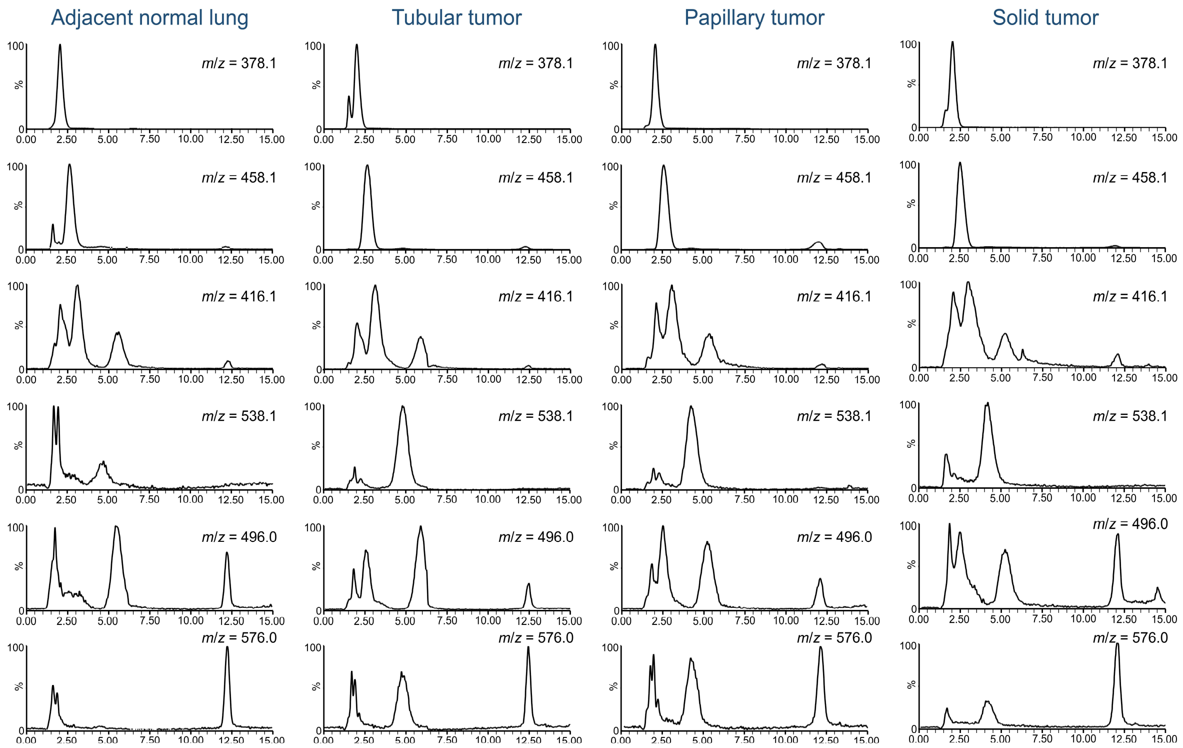

Figure S9. Extracted ion chromatograms of HS disaccharide measurements from regions with different morphological classifications. Figure was created in MassLynx V4.2. The amount of D0A0 was determined by integrating the chromatographic peak areas corresponding to *m*/*z*=378.1 at the retention time of 2 min, D2A0+D0A6 by integrating *m*/*z*=458.1 at 2.5 min, D0S0 by integrating *m*/*z*=416.1 at 3 min, D2A6 by integrating *m*/*z*=538.1 and 576.0 at 4.5 min, D2S0+D0S6 by integrating *m*/*z*=496.0 and 416.1 at 5.5 min, and D2S6 by integrating *m*/*z*= 576.0, 598.0, 496.0 and 416.1 at 12 min.
